# Library strategies differentially shape microbial, functional, and host signals in clinical metagenomic sequencing

**DOI:** 10.1128/msystems.00897-26

**Published:** 2026-09-14

**Authors:** Gengyan Luo, Jin Zhao, Lin-lin Xie, Yirong Li, Yang-zhi Rao, Yanni Sun, Xin Lu, John-Sebastian Eden, Liangjun Chen, Mang Shi

**Affiliations:** 1 National Key Laboratory of Intelligent Tracking and Forecasting for Infectious Diseases, Zhongshan School of Medicine, Shenzhen Campus of Sun Yat-sen University, Sun Yat-sen University, Shenzhen, China; 2 State Key Laboratory for Biocontrol, Zhongshan School of Medicine, Shenzhen Campus of Sun Yat-sen University, Sun Yat-sen University, Shenzhen, China; 3 Department of Laboratory Medicine, Zhongnan Hospital of Wuhan University, Wuhan, China; 4 Hubei Provincial Clinical Research Center for Molecular Diagnostics 624726 , Wuhan, China; 5 Department of Electrical Engineering, City University of Hong Kong, Hong Kong, China; 6 National Key Laboratory of Intelligent Tracking and Forecasting for Infectious Diseases, National Institute for Communicable Disease Control and Prevention, Chinese Center for Disease Control and Prevention, Beijing, China; 7 Sydney Institute for Infectious Diseases, The University of Sydney, Sydney, New South Wales, Australia; 8 Centre for Virus Research, Westmead Institute for Medical Research, Sydney, New South Wales, Australia; 9 Institute of Parasitic Disease Control and Prevention, Center for Disease Control and Prevention of Guangdong Province, Guangzhou, China; The University of British Columbia Okanagan, Kelowna, British Columbia, Canada

**Keywords:** mNGS, total infectome, pathogen detection, functional characterization, host response

## Abstract

Metagenomic next-generation sequencing (mNGS) is increasingly used in infectious disease diagnostics, yet how library preparation shapes the microbial, functional, and host signals recovered from clinical samples remains poorly defined. Here, we performed a within-sample parallel comparison of three mNGS library preparation strategies—DNA-based libraries (DNAlib), RNA-based libraries (RNAlib), and total nucleic acid–based libraries (TNAlib)—across a diverse range of clinical specimens spanning five sample types. Using a curated clinical infectome as a benchmark, we show that library strategies are not interchangeable but capture distinct biological dimensions of the same specimen. RNAlib provided the most comprehensive standalone recovery of the clinical infectome, with improved detection of RNA viruses and cellular pathogens, enhanced resolution of resistance and virulence signals, and preservation of infection-associated host immune signatures. DNAlib showed stronger baseline recovery of DNA viruses and broader host genome coverage, whereas the TNAlib workflow evaluated here largely behaved as an intermediate strategy rather than a consistent improvement over dedicated DNA- or RNA-based workflows. Together, these results establish that the library preparation protocol is a major determinant of how clinical mNGS data should be interpreted and provide a framework for selecting sequencing strategies according to specific diagnostic and biological questions.

**IMPORTANCE:**

Metagenomic sequencing is increasingly used in infectious disease research and clinical diagnostics, but different library preparation strategies may recover fundamentally different biological signals from the same sample. These signals include not only pathogens but also background microbes, microbial functional activity, and host immune-response patterns. Here, we systematically compared DNA-, RNA-, and total nucleic acid–based metagenomic sequencing libraries using the same clinical samples processed in parallel. We found that the three strategies did not provide equivalent information. RNA-based sequencing generated the most informative single-library view of infection, particularly for RNA viruses, cellular pathogens, functional microbial signals, and host immune-response patterns. DNA-based sequencing was more effective for DNA virus and host genome recovery, whereas the total nucleic acid sequencing workflow evaluated here generally behaved as an intermediate strategy. These findings show that library preparation can substantially influence the interpretation of metagenomic data.

## INTRODUCTION

Since metagenomic next-generation sequencing (mNGS) was first used to establish an actionable diagnosis in a case of unexplained neurological infection (1), it has rapidly developed into a hypothesis-free approach for pathogen detection in both research and clinical practice. Over the past decade, mNGS has expanded from proof-of-concept case reports to routine deployment across diverse specimen types and clinical scenarios (2–5). In parallel, its analytical scope has broadened beyond simple pathogen identification to include co-infections, latent or opportunistic microorganisms, and host-associated signals that may inform disease interpretation (6–8). Despite this progress, the clinical application of mNGS remains constrained by substantial methodological heterogeneity, including differences in sample processing, nucleic acid extraction, library preparation, sequencing depth, and bioinformatic workflows (2, 9–15). These variables can markedly affect both detection sensitivity and quantitative signal structure, thereby limiting reproducibility across studies and complicating biological and clinical interpretation.

Among these variables, library preparation is particularly important because it determines which nucleic acid molecules enter the sequencing pool and therefore directly shapes both detectability and downstream signal composition (14, 16, 17). In current practice, most workflows adopt either DNA-based or RNA-based strategies. DNA-based mNGS generally provides robust recovery of DNA-derived molecules and is well suited for broad genomic characterization of pathogens (18–20). By contrast, RNA-based mNGS enriches RNA-derived molecules, improves recovery of RNA viruses, and provides access to biologically active microbial and host transcriptional signals (6, 21–23). More recently, total nucleic acid–based workflows have been proposed as a unified strategy intended to capture both DNA and RNA within a single library preparation (24–28). These approaches are increasingly applied in clinical settings, yet their relative strengths and limitations remain incompletely defined.

Existing studies suggest that different library strategies offer distinct and partly complementary advantages. DNA-based workflows may favor recovery of DNA pathogens, whereas RNA-based workflows may better capture transcriptionally active organisms and improve discrimination between infection and colonization in some settings (22, 29–32). This has led to growing interest in either combined DNA/RNA sequencing or unified total nucleic acid workflows as practical solutions for complex or undiagnosed infections (22, 24–28, 30, 32–34). However, current comparative evidence remains limited in several important respects. First, many studies have been restricted to specific syndromes, specimen types, or small cohorts. Second, direct comparisons are often confounded by non-parallel study designs, making it difficult to separate genuine library-specific effects from inter-sample heterogeneity or reagent-derived background contamination (9, 35). Third, benchmarking has largely focused on detection of a single dominant or clinically reported pathogen, whereas clinical samples often contain a broader spectrum of microorganisms, including co-infecting pathogens, opportunists, and latent viruses, all of which may influence disease presentation and interpretation (36). A more systematic framework is therefore needed to assess how library chemistry shapes the multidimensional “clinical infectome” captured by mNGS.

Beyond organismal detection, the functional and host-derived information embedded in mNGS data is also likely to depend strongly on library design. RNA-based workflows may preferentially capture transcriptionally active processes, including microbial virulence programs, antibiotic resistance gene expression, and host immune responses, whereas DNA-based workflows more directly represent genomic content and organismal burden (37–40). However, these differences have not been systematically evaluated across diverse clinical specimen types under a strictly controlled, within-sample design. Such knowledge is essential not only for improving interpretation of mNGS data but also for selecting the most appropriate sequencing strategy for different diagnostic and biological questions.

Here, we performed a rigorous within-sample parallel comparison of three mNGS library strategies—DNA-based (DNAlib), RNA-based (RNAlib), and total nucleic acid–based (TNAlib)—across 96 clinical specimens representing 5 major sample types. Using a unified experimental workflow with parallel process controls, we defined a high-resolution clinical infectome as a benchmark and systematically evaluated how library preparation influences microbial detection, functional profiling of antibiotic resistance genes and virulence factors, and host information recovery. By resolving these library-dependent effects across microbial, functional, and host-response dimensions, our study provides an evidence-based framework for selecting sequencing strategies in clinical mNGS and for interpreting the biological signals captured by different library chemistries.

## RESULTS

### Clinical cohort and within-sample parallel design for mNGS library comparison

**Fig 1:**
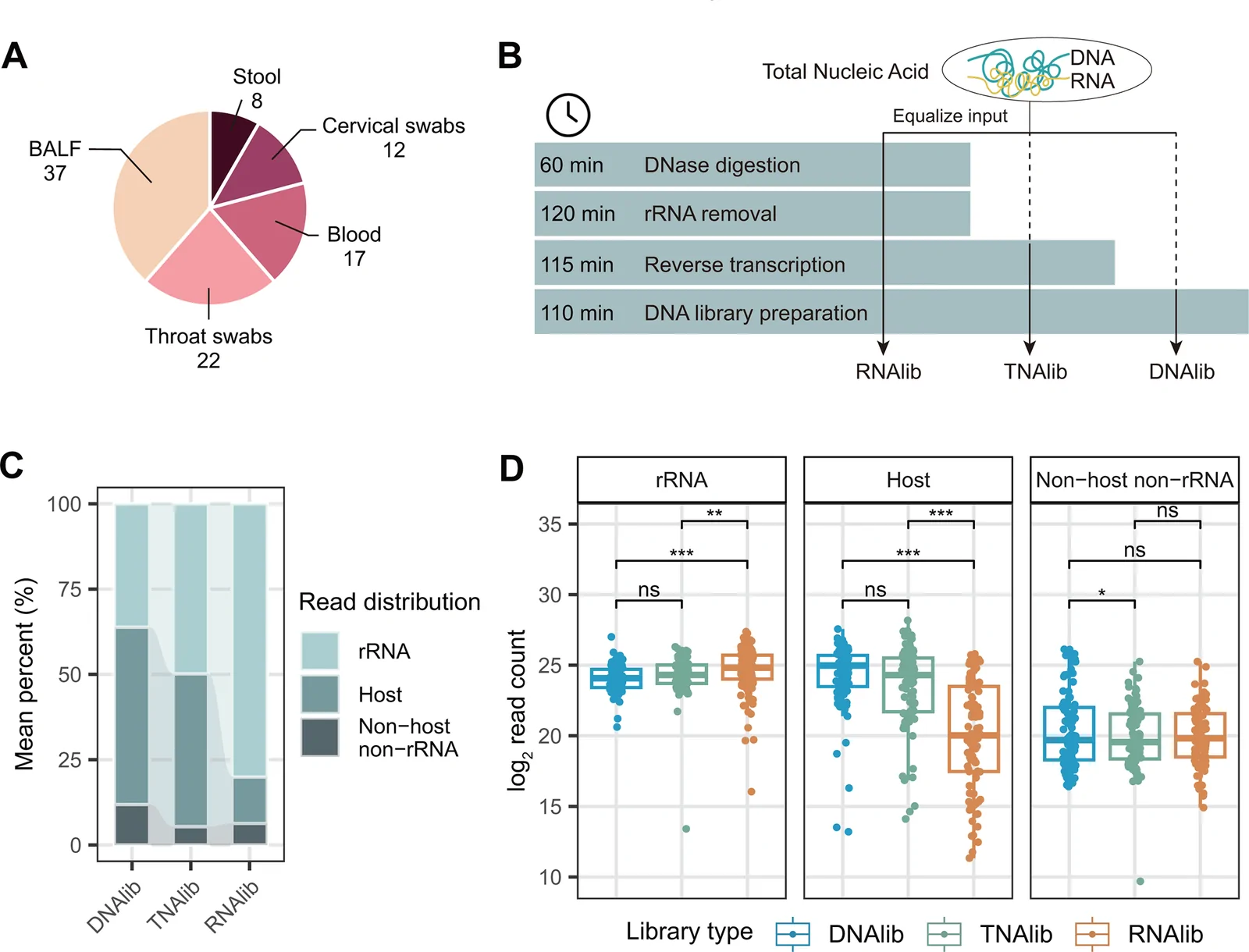
Overview of clinical specimens, library workflows, and read composition. (**A**) Distribution of 96 clinical specimens across five sample types. (**B**) Parallel library construction workflow. Following total nucleic acid (TNA) extraction, equal input amounts from each sample were allocated to RNA-based (RNAlib), total nucleic acid-based (TNAlib), and DNA-based (DNAlib) library construction. Approximate processing times for the major workflow steps are shown on the left. (**C**) Mean proportional composition of sequencing reads after preprocessing, partitioned into rRNA, host-derived, and non-host/non-rRNA reads for each library type. (**D**) Absolute numbers of rRNA, host-derived, and non-host/non-rRNA reads after preprocessing, shown as log₂-transformed read counts. Center lines indicate medians, and boxes represent interquartile ranges (IQRs). Statistical significance was assessed using paired Wilcoxon signed-rank tests with Benjamini–Hochberg correction and is indicated as ns, not significant; *P < 0.05; **P < 0.01; and ***P < 0.001.

We analyzed 96 clinical specimens representing 5 common clinical sample types—bronchoalveolar lavage fluid (BALF), throat swabs, blood, cervical swabs, and stool—to enable a systematic comparison of mNGS library construction strategies under conditions reflecting routine clinical practice (Fig. 1A; Table S1). The cohort encompassed diverse clinically reported infection categories, including bacterial, fungal, DNA viral, RNA viral, and retro-transcribing viral infections, as well as 21 specimens without prior etiological testing (“Not assessed”), including all stool specimens (Table S1).

Each specimen was subjected to a strict within-sample parallel design, in which identical total nucleic acid inputs were evenly allocated to construct three library types: RNAlib, TNAlib, and DNAlib (Fig. 1B). This design minimized confounding effects from inter-sample variability and enabled direct, head-to-head comparison of library strategies across the same biological background. Workflow-level comparisons further showed stepwise increases in reagent costs and processing time from DNAlib to TNAlib and RNAlib, providing practical context for evaluating the feasibility and implementation requirements of each library strategy (Table S2).

To characterize background signals introduced by reagents and laboratory workflows, 15 process control (PC) libraries were prepared in parallel (3 per sample type), resulting in a total of 111 sample inputs processed and sequenced under identical conditions. All libraries were sequenced using a unified high-throughput sequencing protocol. Among clinical samples, the median sequencing yields were 70.9 million reads for DNAlib, 68.3 million reads for TNAlib, and 58.5 million reads for RNAlib, providing comparable sequencing depth across library types and ensuring robust downstream performance evaluation. Read partitioning after preprocessing revealed distinct library-specific profiles in both relative composition and absolute read counts (Fig. 1C and D). DNAlib contained the highest proportion and number of host-derived reads, whereas RNAlib retained the greatest ribosomal RNA (rRNA) burden. After removal of host and rRNA reads, the number of non-host/non-rRNA reads available for downstream microbial profiling was broadly comparable between DNAlib and RNAlib but lower in TNAlib (Fig. 1D).

### Defining the clinical infectome as a benchmark for mNGS-based pathogen detection

**Fig 2:**
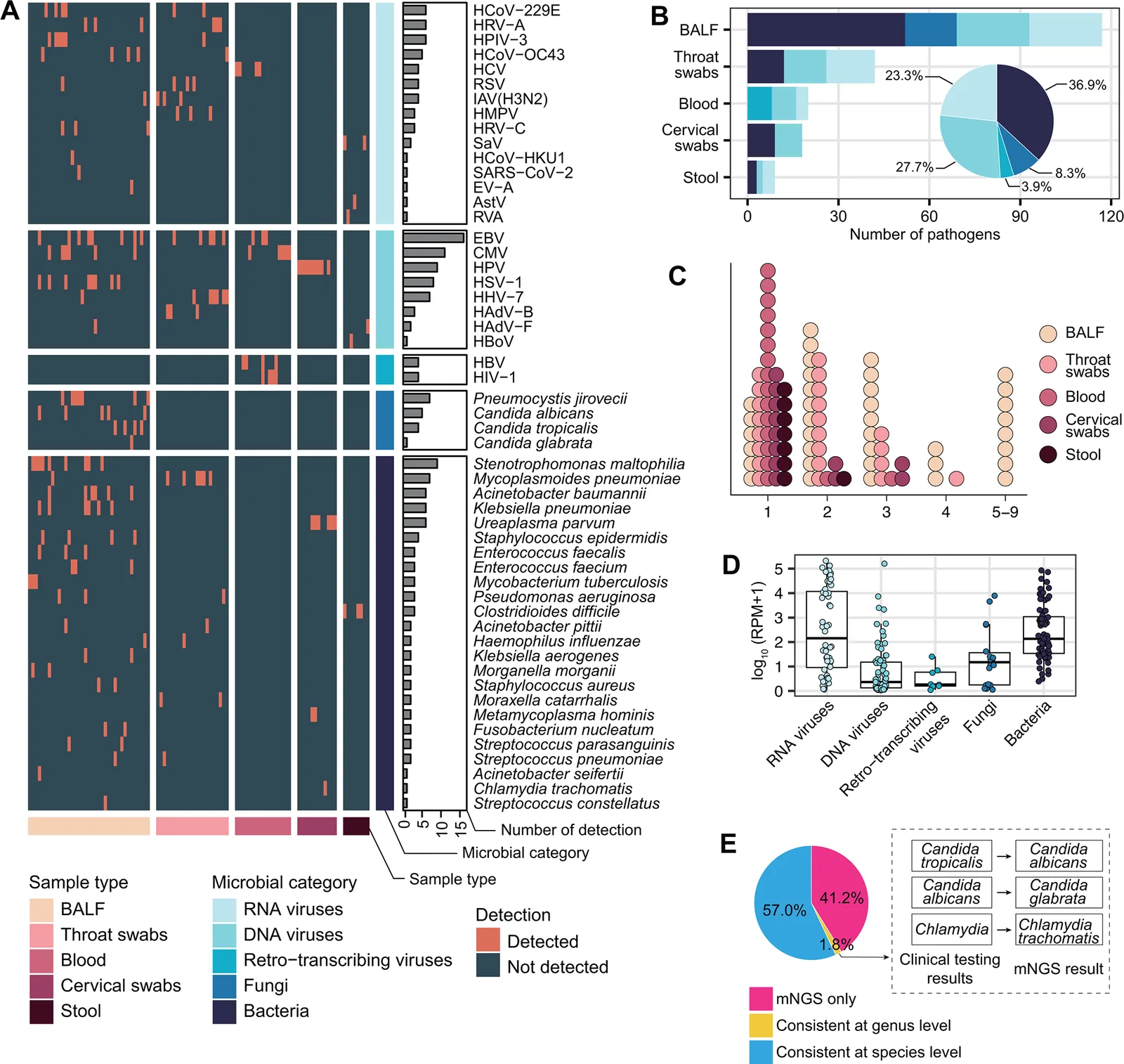
Composition, distribution, and concordance of the infectome with clinical testing results across the clinical cohort. (**A**) Overview of the infectome landscape across 96 clinical samples. (**B**) Distribution of infectome detections across specimen types. Stacked bar plots indicate the number of infectome detections within each sample type, stratified by microbial category. (**C**) Number of infectome species detected per sample. Each dot represents an individual sample, grouped by sample type. (**D**) Infectome abundance across microbial categories. Boxplots show reads per million (RPM), defined as the maximum RPM obtained across the three library types (DNAlib, TNAlib, and RNAlib) for each infectome detection. (**E**) Agreement between mNGS detections and clinical testing results after excluding specimens classified as “Not assessed.”

All 96 clinical samples underwent species-level microbial identification to define the clinical infectome, conceptualized as the ensemble of microorganisms with potential relevance to infection. Phylogenetic analyses further supported the accuracy of these taxonomic assignments (Fig. S1 and S2). The curated infectome comprised 53 distinct species, including 15 RNA viruses, 8 DNA viruses, 2 retro-transcribing (RT) viruses, 4 fungi, and 24 bacterial species, collectively spanning 5 microbial categories and a broad spectrum of clinically relevant pathogens (Fig. 2A; Table S3). When benchmarked against this curated infectome, Kraken2 recovered 79.1% of all infectome detections, whereas MetaPhlAn4 recovered 76.3% of bacterial infectome detections (Fig. S3A). Neither classifier identified additional pathogens that could be independently confirmed (Fig. S3B and C).

The infectome encompassed diverse pathogenic categories, including obligate pathogens (e.g., influenza A viruses, HIV, and *Mycobacterium tuberculosis*), opportunistic pathogens (e.g., rhinoviruses and *Candida albicans*), multidrug-resistant nosocomial bacteria (e.g., *Acinetobacter baumannii* and *Klebsiella pneumoniae*), as well as less frequently reported pathogens (e.g., human bocavirus and sapovirus). Across the five microbial categories, DNA viruses and bacteria represented the most abundant and widely distributed components (Fig. 2B). Herpesviruses were among the most frequently detected taxa, with positivity rates of 47.1% in blood, 40.5% in BALF, and 36.4% in throat swabs (Table S1). Multiple-pathogen detections were common, with 54.2% of samples containing at least two pathogens and 8.3% containing at least five. These detections were most frequent in BALF and throat swab specimens (Fig. 2C). Infectome abundance was substantial, with a median of 24.3 reads per million (RPM) and peak values reaching 10^6^ RPM (up to 54.8% of total non-rRNA reads) (Fig. 2D).

Comparison with routine clinical test results, including PCR, culture, or external clinical mNGS testing, showed only partial concordance at the species level. After excluding specimens classified as “Not assessed,” 57.0% of mNGS detections were consistent with the reported clinical findings, whereas 41.2% were not detected or were assigned differently by routine testing, and 1.8% showed concordance only at the genus level (Fig. 2E; Table S1). These discrepancies likely reflect the limited taxonomic resolution of conventional diagnostic assays. Collectively, the breadth, abundance, and species-level resolution of the infectome defined here establish a robust benchmark for systematic evaluation of mNGS library preparation strategies.

### Defining the optimal sequencing strategy for comprehensive infectome profiling

**Fig 3:**
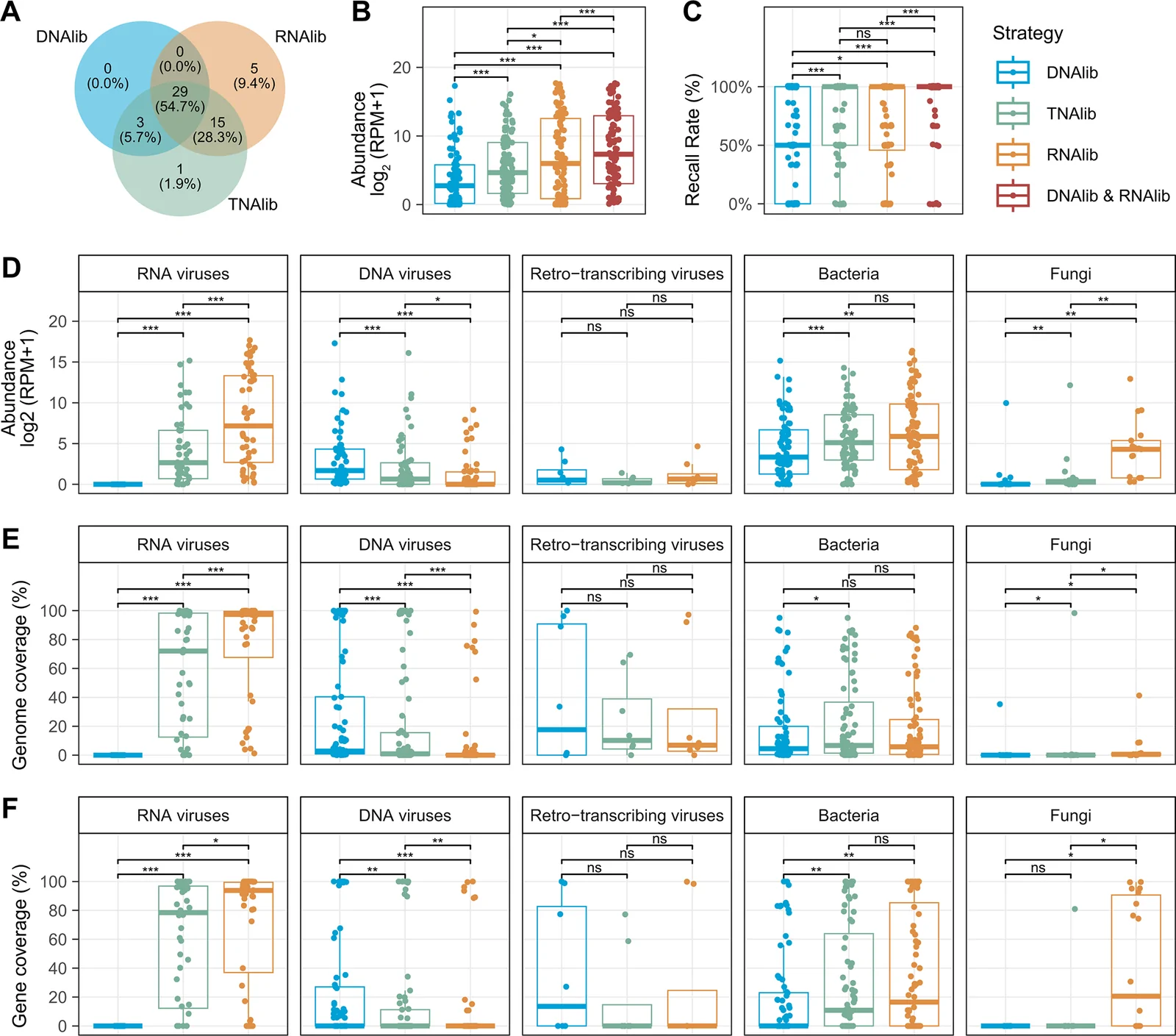
Multi-level benchmarking of infectome detection across library strategies. (**A**) Venn diagram showing the overlap of infectome species detected by DNAlib, TNAlib, and RNAlib across the clinical cohort. Numbers and percentages indicate species counts and their fractions relative to the total infectome species set. (**B**) Sample-level infectome abundance across four library strategies—DNAlib, TNAlib, RNAlib, and the combined DNAlib and RNAlib strategy—shown as log₂(RPM + 1), where RPM represents the total RPM across all infectome species detected in each sample. (**C**) Sample-level recall rate across four library strategies, calculated as the fraction of benchmark infectome species recovered in each sample. (**D**) Species-level infectome abundance across library types, stratified by microbial category, shown as log₂(RPM + 1). (**E and F**) Genome coverage (**E**) and conserved marker gene coverage (**F**) for infectome detections across library types, stratified by microbial category. In boxplots **B –F**, center lines indicate medians and boxes indicate interquartile ranges (IQRs). Points represent individual samples in panels **B and C** and individual detections in panels **D–F**. Statistical significance was assessed using paired Wilcoxon signed-rank tests with Benjamini–Hochberg (BH) correction for multiple comparisons and is annotated as ns (not significant), *P < 0.05, **P < 0.01, and ***P < 0.001.

To determine the most effective strategy for comprehensive infectome profiling, we compared three library types across the clinical cohort. RNAlib recovered the largest fraction of infectome species (92.5%), followed by TNAlib (90.6%) and DNAlib (60.4%), and generated the strongest quantitative signals and highest recall rates overall (Fig. 3A through C). Although TNAlib simultaneously captures RNA and DNA, it did not outperform RNAlib in either diversity or abundance and ranked second overall. Notably, separating RNAlib and DNAlib at the sample level and integrating their outputs further improved spectrum coverage, recall, and abundance estimates, surpassing RNA sequencing alone (Fig. 3A through C). Sample type–stratified analyses showed that the main library-specific trends observed in the pooled cohort were broadly preserved across specimen types, with RNAlib generally providing the most comprehensive infectome recovery (Fig. S4). However, several within-type comparisons did not reach statistical significance, likely because of the limited sample sizes in individual strata. These results position RNAlib as the most effective standalone strategy, while combined sequencing maximizes overall infectome resolution.

Stratification by microbial category revealed clear, method-specific performance differences. RNAlib preferentially enhanced detection of RNA viruses, bacteria, and fungi, whereas DNAlib showed stronger quantitative signals for DNA viruses (Fig. 3D, Fig. S5). Under the default 0.5 RPM cutoff, DNAlib and TNAlib achieved complete DNA virus recovery (8/8), whereas RNAlib recovered five species. However, this apparent disadvantage proved highly threshold-dependent. Reducing the RPM cutoff from 0.5 to 0.1 substantially increased DNA virus recall in RNAlib (from 24.0% to 36.0%; Fig. S5), narrowing the performance gap. These microbe-specific patterns were further illustrated by representative species-level abundance examples (Fig. S6). Genome and conserved marker gene coverage largely paralleled abundance patterns (see Materials and Methods for the conserved marker genes analyzed) (Fig. 3E and F). Notably, for cellular pathogens, RNAlib achieved deeper marker gene coverage than DNAlib while maintaining comparable genome-level coverage (Fig. 3E and F).

Together, these results demonstrate that infectome profiling is both library-dependent and threshold-sensitive, with RNAlib favoring RNA viruses and cellular microbes, DNAlib providing stronger default DNA virus recovery, and optimal strategy selection contingent on both library design and cutoff calibration.

### Library-dependent functional landscapes of the infectome

**Fig 4:**
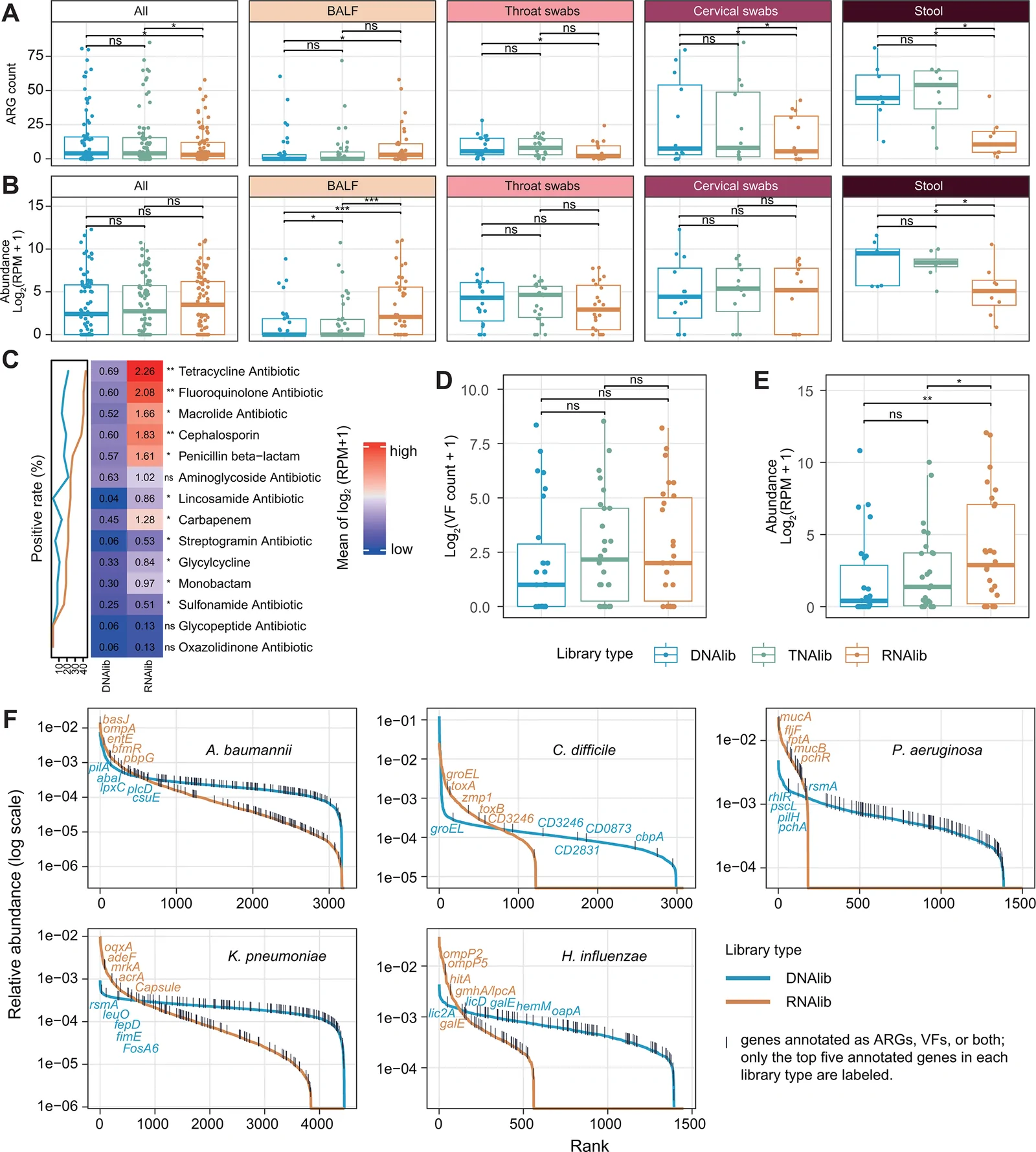
Library-dependent functional landscapes of resistance and virulence genes. (**A**) Numbers of ARGs detected across DNAlib, TNAlib, and RNAlib in all non-blood samples (*n* = 79), stratified by sample type. (**B**) Total abundance of detected ARGs across the same samples, stratified by sample type, shown as log₂(RPM + 1), where RPM represents the summed abundance of detected ARGs per sample. (**C**) Comparison of ARG-associated antibiotic class annotations between DNAlib and RNAlib in BALF samples based on 14 representative antibiotic classes (see Materials and Methods for details). Line plots show the detection rate of each class, and the heatmap shows the mean abundance of detected ARG annotations associated with each class [mean log₂(RPM + 1)]. Significance annotations next to antibiotic class labels indicate DNAlib–RNAlib differences. (**D**) Numbers of VFs detected for DNAlib, TNAlib, and RNAlib in the subset of samples containing bacteria with annotated VFs (*n* = 26). (**E**) Total abundance of detected VFs in the same subset of samples, shown as log₂(RPM + 1), where RPM represents the summed abundance of detected VFs. (**F**) Pathogen-level rank–abundance distributions comparing DNAlib and RNAlib for representative bacterial pathogens (*A. baumannii*, *K. pneumoniae*, *P. aeruginosa*, *Haemophilus influenzae*, and *C. difficile*). Relative gene abundance is shown on a log scale. Vertical tick marks indicate the positions of genes detected and annotated as ARGs, VFs, or both, and labels are shown only for the five most abundant annotated genes in each library type. In box plots **A, B, D, and E**, center lines indicate medians and boxes indicate interquartile ranges (IQRs); points represent individual sample values. Statistical significance was assessed using paired Wilcoxon signed-rank tests with Benjamini–Hochberg (BH) correction for multiple comparisons and is annotated as ns (not significant), *P < 0.05, **P < 0.01, and ***P < 0.001.

To evaluate how sequencing strategy influences functional characterization of the infectome, we compared antibiotic resistance genes (ARGs) and virulence factors (VFs) across strategies, excluding blood samples (*n* = 79) due to their typically sterile nature. At the sample level, RNAlib detected fewer ARGs per sample (median = 3) than DNAlib and TNAlib (median = 4 each) (Fig. 4A), although the total abundance of detected ARGs per sample was comparable across three library types (Fig. 4B). Stratification by sample type revealed context-dependent effects. In BALF samples, RNAlib detected more ARGs and showed a higher total abundance of detected ARGs than DNAlib and TNAlib (Fig. 4A and B). In contrast, for non-BALF specimens, RNAlib consistently detected fewer ARGs than DNAlib, while the total abundance of detected ARGs remained broadly similar across library types except in stool samples.

At the functional annotation level, comparison of 14 representative antibiotic classes, spanning broad-spectrum empirical options and critical salvage therapies (Table S4), revealed limited but context-specific differences. In BALF samples, RNAlib showed higher abundances of detected ARGs and higher ARG detection rates for commonly used empirical and targeted classes, including tetracyclines, fluoroquinolones, macrolides, and beta-lactams (Fig. 4C). Outside BALF, class-level differences between DNAlib and RNAlib were modest and inconsistent, with DNAlib showing slightly higher detection rates for several classes (Fig. S7).

For virulence factors (VFs), analyses were performed at the sample level and restricted to samples in which bacteria carrying annotated VFs were detected (*n* = 26). The VF repertoire was dominated by major pathogenic mechanisms, including effector delivery systems (e.g., the *Pseudomonas aeruginosa* type VI secretion system and *M. tuberculosis* ESX complexes), potent exotoxins (e.g., *Clostridioides difficile* TcdA/TcdB), and adherence factors such as type IV pili. Although the number of VFs detected did not differ significantly across library strategies (Fig. 4D), RNAlib yielded significantly higher total abundance of detected VFs than DNAlib and TNAlib (Fig. 4E).

Importantly, integrative analysis of ARGs and VFs at the pathogen level revealed systematic differences in functional signal structure. RNAlib exhibited a broader dynamic range in the relative abundance distributions of both detected ARGs and VFs, highlighting dominant resistance and virulence contributors, whereas DNAlib showed more compressed abundance profiles (Fig. 4F). Collectively, these results demonstrate that library strategy shapes functional profiling in a context- and microbe-dependent manner. While the number of ARGs detected varied by specimen type, RNAlib enhances quantitative resolution of both resistance and virulence signals, suggesting greater sensitivity to transcriptionally active functional elements.

### Reduced host genome recovery but preserved infection-specific transcriptomic resolution under RNAlib

To assess host information recovery across library strategies, we first compared host read abundance across all 96 clinical samples. DNAlib and TNAlib yielded substantially higher host RPM than RNAlib (median 8.6-fold and 6.4-fold increases in host RPM, respectively; both BH-adjusted *P* < 0.001; Fig. S8A). Linear mixed-effects modeling further showed that genome coverage increased more efficiently with host RPM in DNAlib and TNAlib than in RNAlib (Fig. S8B; both BH-adjusted *P* < 0.001 for DNAlib–RNAlib and TNAlib–RNAlib slope contrasts), indicating superior host genome recovery under DNA-based approaches. These trends were consistent across specimen types, with only modest variation attributable to sample type (Fig. S8C).

**Fig 5:**
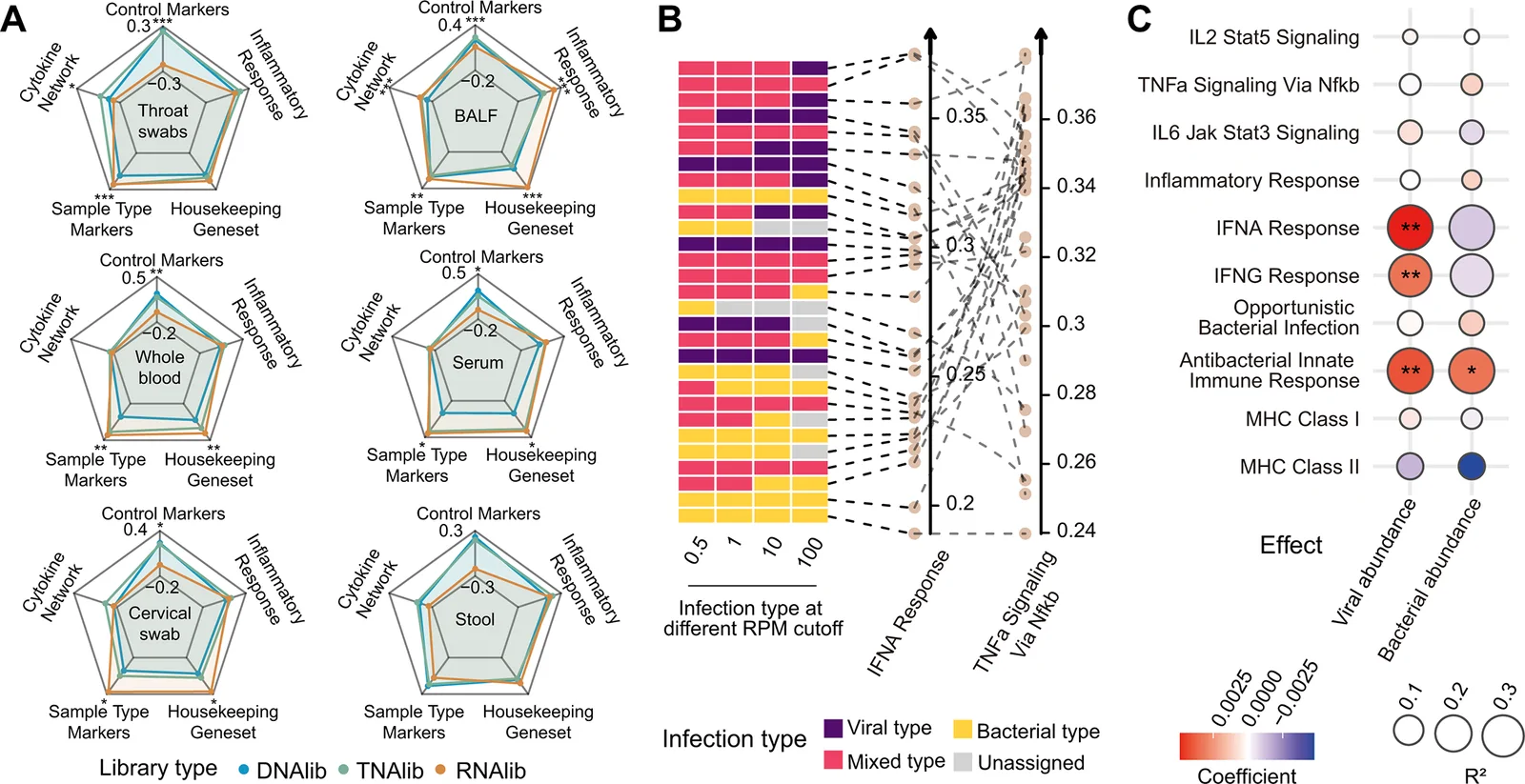
Host transcriptomic responses link pathogen load to infection-specific immune signatures. (**A**) Radar plots summarizing activity of five representative gene sets across library types. For each specimen type, polygons show the median singscore for each gene set, with colors indicating library type. Asterisks next to gene set labels indicate statistical significance for the paired DNAlib–RNAlib comparison (*P < 0.05, **P < 0.01, ***P < 0.001; paired Wilcoxon signed-rank tests with Benjamini–Hochberg correction). (**B**) Infection-type assignment and immune-response gene-set scores in BALF samples with sufficient host read depth (*n* = 32) under different RPM cutoffs. The left heatmap shows infection-type classification (viral type, mixed type, bacterial type, or unassigned) at RPM cutoffs of >0.5, >1, >10, and >100, using an optimal RPM definition (DNA viruses from DNAlib; other microbes from RNAlib). The right panel shows singscores for IFNα response and TNFα signaling via NFκB, with points colored by gene set. Dashed lines link the infection-type assignment to the corresponding gene-set singscores for the same sample. (**C**) Bubble plot summarizing regression analyses linking pathogen abundance to host gene-set singscores in BALF samples with sufficient host read depth (*n* = 32). For each gene set, linear models were fitted with singscores as the response and bacterial and viral abundance [log₂(RPM + 1)] as predictors. Bubble size denotes model *R*², and fill color denotes the regression coefficient. Asterisks indicate Benjamini–Hochberg adjusted significance of regression coefficients (*P < 0.05, **P < 0.01, ***P < 0.001).

Despite reduced host genomic breadth, RNAlib retained distinct and biologically informative transcriptomic patterns. Gene set–level analyses in a subset with sufficient host read depth (*n* = 71) revealed selective enrichment rather than uniform signal loss relative to DNAlib (Fig. 5A). For RNAlib, housekeeping genes and sample type–specific gene sets were relatively preserved across most specimens, suggesting maintenance of core cellular functions. More importantly, immune-related pathways—including inflammation-associated signatures (BALF) and cytokine network genes (BALF)—were preferentially enriched in RNAlib, indicating enhanced capture of transcriptionally active host responses in relation to infection.

Focusing on BALF samples, we further evaluated infection type–specific host signatures. At an intermediate pathogen load threshold (10 RPM), gene set scores aligned with infection classification: bacterial infections exhibited higher TNFα signaling via NFκB, viral infections showed elevated IFNα response, and mixed infections demonstrated activation of both pathways (Fig. 5B). Additional gene sets showed limited separation among infection groups under this threshold (Fig. S8D). Regression analyses confirmed quantitative coherence, with antiviral gene sets positively associated with viral load and antibacterial gene sets associated with bacterial load (Fig. 5C).

Together, these findings indicate that although RNAlib provides narrower host genome recovery, it effectively captures pathogen-associated immune activation and preserves quantitative coupling between pathogen burden and host transcriptional response.

## DISCUSSION

Metagenomic next-generation sequencing (mNGS) is increasingly used as a universal diagnostic framework capable of capturing microbial diversity, functional activity, and host responses in a single experiment. However, how library preparation strategies shape these multidimensional signals remains insufficiently understood. Here, using a strictly controlled within-sample parallel design, we compared three commonly used library preparation workflows—DNAlib, RNAlib, and TNAlib—across diverse clinical specimens to systematically evaluate the impact of library chemistry on mNGS outputs. Our analyses reveal three key insights. First, among standalone workflows, RNAlib provided the most comprehensive recovery of the clinical infectome, generating the strongest microbial signals while also capturing transcriptionally active microbial functions and infection-associated host responses. Second, DNAlib showed stronger baseline recovery of DNA viruses and captured a substantially broader fraction of host genomic material. Third, the TNAlib workflow evaluated here performed largely as an intermediate strategy: it underperformed DNAlib in DNA virus detection and RNAlib in recovery of other microbial taxa, while providing limited functional or host-response information. Together, these findings clarify the biological trade-offs underlying clinical mNGS workflows and provide a systematic benchmark for interpreting multidimensional sequencing data.

Viewed through the sample-level framework, our results suggest that the widespread use of DNA-based sequencing as the default strategy for undiagnosed infections warrants reconsideration. Previous comparisons of mNGS workflows have often focused on analytical sensitivity for predefined pathogens (22, 30, 32). In clinical practice, however, the relevant question is which strategy best captures the full infectome present within a specimen. Under this framework, RNAlib emerged as the most informative standalone approach. This advantage likely reflects a fundamental biological distinction between analytes: DNA sequencing primarily captures genomic presence, whereas RNA sequencing more directly reflects transcriptional activity and is therefore more closely aligned with pathogen activity *in vivo* (41). Consistent with this distinction, RNAlib enriched highly expressed microbial signals, provided deeper coverage of phylogenetically informative marker genes in cellular pathogens, and maximized recovery of RNA viruses, albeit with reduced baseline sensitivity for transcriptionally silent DNA viruses. These analytical advantages must, however, be balanced against practical limitations, including higher reagent costs, additional processing steps, and the greater susceptibility of RNA to degradation. Moreover, RNAlib uniquely incorporated an rRNA depletion step, whereas DNAlib and TNAlib did not undergo an equivalent depletion procedure. Its improved microbial recovery should therefore be attributed to the combined effects of RNA-based profiling and workflow-specific enrichment, rather than to RNA sequencing alone.

The key issue is that higher default recovery of DNA viruses does not necessarily translate into greater clinical relevance. DNAlib showed stronger DNA virus recovery at default analytical thresholds, largely driven by low-abundance herpesvirus detections. However, this apparent advantage should not be taken as evidence of superior diagnostic performance. Many DNA viruses, particularly herpesviruses and polyomaviruses, can persist in latent or low-transcription states, such that DNA sequencing may capture genomic persistence or background shedding rather than active infection (42–44). By contrast, RNAlib is intrinsically biased toward transcriptionally active organisms and therefore under-recovers transcriptionally silent DNA viruses unless analytical thresholds are relaxed. Consistent with this interpretation, lowering the RNA-based detection cutoff partially improved DNA virus recall in our data set, suggesting that the observed differences reflect not only library strategy but also the biological state of the detected viruses.

Our results also caution against assuming that the TNAlib workflow evaluated here necessarily provides an optimal all-in-one solution. In our implementation, TNAlib generally behaved as an intermediate strategy rather than a consistent improvement over dedicated DNA- or RNA-based libraries. This pattern likely reflects the inherent challenge of capturing multiple molecular pools within a single workflow, including abundant host genomic DNA and structural RNA species, which may reduce effective sequencing capacity for low-abundance microbial signals (45, 46). In addition, by combining DNA- and RNA-derived molecules into the same library, TNAlib may compromise the interpretability of each signal type, making it less well suited for clearly resolving either genomic burden or transcriptional activity. Taken together, our data suggest that, although TNAlib offers practical advantages by consolidating workflows, the workflow tested in this study should be viewed primarily as a convenience-oriented compromise rather than a universally superior strategy, and its use is most justified when logistical simplification is a priority.

Library-dependent signal partitioning was also evident in functional profiling of ARGs and VFs. DNAlib primarily reflects the genomic repertoire of resistance and virulence determinants, whereas RNAlib reflects the subset represented by RNA transcripts at the time of sampling. Therefore, ARGs or VFs that are present in microbial genomes but are not transcribed, or are expressed at very low levels, may be missed or under-represented in RNAlib. Comparisons between DNA- and RNA-based libraries should therefore be interpreted as differences between genomic potential and transcriptional representation, rather than as equivalent measures of gene presence. These differences were particularly apparent in respiratory specimens, where RNA-based profiling more clearly highlighted infection-associated functional programs than in heavily colonized niches such as the gut, where the underlying functional reservoir remained relatively stable across workflows. These functional landscapes should nevertheless be interpreted cautiously. Because clinical mNGS is typically read-based and applied to host-dominated specimens, it cannot reliably resolve genomic linkage or strain-level context for individual ARGs and VFs (47, 48). RNA-based sequencing does not overcome this limitation, but it provides an important complementary layer by identifying which resistance and virulence programs are actively expressed (49).

Host-derived signals represent another important dimension shaped by library strategy. Although host reads are often regarded as an analytical burden in infection-focused mNGS workflows and are removed in many protocols (10, 50), our results show that RNAlib preserves a clinically informative host transcriptomic layer while substantially reducing genomic background. In our data set, this layer retained coherent inflammatory and cytokine-related programs and remained quantitatively coupled to pathogen burden, indicating that RNA-based sequencing captures biologically active host responses rather than merely residual host material. This observation is consistent with previous studies showing that host transcriptional signals derived from mNGS can substantially improve interpretation of infectious syndromes. For example, Langelier et al. (6) demonstrated that integrating host response signatures with unbiased microbial detection improved discrimination of lower respiratory tract infection from non-infectious etiologies in critically ill adults. Similarly, Kalantar et al. (7) showed that host–microbe plasma metagenomics could enhance diagnostic resolution in sepsis by combining pathogen detection with host-response patterns. Together, these studies support the view that host information, especially transcriptional signal from RNAlib, is not simply an accessory output, but a biologically and clinically meaningful signal layer.

These methodological differences also have important implications for downstream computational analysis. As clinical mNGS data sets are increasingly used for machine-learning and foundation-model applications (51–53), library construction should not be regarded merely as a technical covariate but as a determinant of the biological signal space available for modeling. In particular, our study reveals that RNAlib captures a richer and more layered representation of the specimen, simultaneously reflecting microbial gene sequences and activities, functional gene expression, and host immune responses. This multidimensional signal structure is likely to be especially valuable for computational models aimed at resolving infection status, pathogen activity, and host–microbe interactions. Accordingly, explicit consideration of library-dependent signal composition will be essential for developing robust, context-aware models that can generalize across heterogeneous clinical mNGS data sets.

Our findings further support the potential value of parallel DNA and RNA sequencing from the same specimen. In our cohort, this dual-library strategy improved overall infectome recovery and added an important interpretive dimension for functional profiling. RNA sequencing captures dynamically expressed microbial programs, while DNA sequencing provides the genomic reservoir against which transcriptional activity can be interpreted (54, 55). Integrating these two analytes therefore offers a more informative framework for distinguishing active microbial processes from genomic persistence. At the same time, practical considerations remain important. RNAlib is currently associated with greater workflow complexity and potentially higher costs than DNAlib (56), which likely contributes to its more limited adoption in routine settings, and parallel DNA–RNA sequencing further increases overall expense. These cost constraints may limit immediate implementation, although they are likely to lessen as sequencing technologies and library workflows continue to mature.

Several limitations should be considered. First, because the clinical infectome benchmark incorporated sequencing evidence generated in this study, benchmark-based comparisons across library strategies may be affected by some degree of circularity, particularly as culture- or PCR-based confirmation was not available for every sample or every detected pathogen owing to limitations in sample availability and routine testing scope. Second, as a single-center study, our cohort may not fully capture the epidemiological diversity of clinical infectomes across geographic regions and patient populations. Third, although our within-sample parallel design enabled controlled comparisons, the cohort size remained modest, limiting statistical power for rare pathogens and specific infection subtypes. Finally, our findings should be interpreted in the context of the specific workflow evaluated here. Although the same total nucleic acid extraction protocol was used for all three library types, differences in extraction chemistry, lysis efficiency, microbial enrichment, library preparation kits, and sequencing platforms may affect microbial recovery. Therefore, the performance patterns observed here may not fully generalize to other mNGS workflows.

In summary, our within-sample parallel comparison shows that library preparation is a major determinant of the biological information recovered by clinical mNGS. DNAlib, RNAlib, and TNAlib are therefore not interchangeable but capture different and partially overlapping features of the same specimen. Among them, RNAlib provided the most informative standalone view of active infection by better preserving microbial, functional, and host-response signals, whereas TNAlib behaved mainly as a practical compromise in our implementation. These findings provide a framework for selecting library strategies according to specific clinical and biological goals and for more accurate interpretation of multidimensional mNGS data.

## MATERIALS AND METHODS

### Sample collection and total nucleic acid extraction

Clinical specimens were collected from outpatients and hospitalized patients with suspected infection at Zhongnan Hospital of Wuhan University between 2022 and 2024 as part of routine diagnostic testing. Immediately after collection, samples were transferred into RNA*later* (Invitrogen, USA) and stored at −80 °C until processing. Total nucleic acids (TNAs) were extracted using the Nucleic Acid Extraction or Purification Reagents (DAAN GENE, China) according to the manufacturer’s instructions, and DNA and RNA concentrations were quantified using a Qubit 4.0 Fluorometer (Invitrogen, USA).

Process control (PC) samples were generated for each specimen type to characterize background signals introduced by reagents and sampling devices. Sterile saline was passed through the corresponding collection devices, followed by TNA extraction using the same workflow applied to clinical samples. Extracts were then spiked with DNA and RNA standards (control components of the TruePrep Flexible DNA Library Prep Kit, Vazyme, China, and the Trio RNA-Seq Kit, TECAN, Switzerland) to match average nucleic acid concentrations measured in clinical samples of the corresponding specimen type.

### Parallel library preparation and sequencing

For each specimen, TNA input volumes were adjusted based on RNA concentration to provide 10 ng RNA per library (maximum input volume 16.5 μL for low-biomass samples). Three libraries were constructed in parallel from each extract: RNA-based libraries (RNAlib), total nucleic acid libraries (TNAlib), and DNA-based libraries (DNAlib). RNAlib preparation involved DNase I (Vazyme, China) digestion followed by rRNA depletion using Ribo-off rRNA Depletion Kit (Vazyme, China) and reverse transcription using double-stranded complementary DNA (cDNA) Synthesis Module2 Kit (Vazyme, China) to generate double-stranded cDNA prior to DNA library construction using TruePrep Flexible DNA Library Prep Kit (Vazyme, China). TNAlib libraries were generated by reverse transcription of TNA extracts followed by the same DNA library preparation workflow. DNAlib libraries were constructed directly from TNA extracts without reverse transcription. Libraries were quality-checked using Qsep1 (Bioptic, China) and sequenced on an Illumina NovaSeq platform (Illumina, USA) in a paired-end 150 bp mode, targeting ~10 Gb of sequencing data per library.

### Read preprocessing

Sequencing reads were preprocessed to remove technical artifacts, rRNA reads, and host-derived reads prior to downstream analyses. Adapter trimming and low-complexity filtering were performed using BBMap (57) (v38.62), followed by de-duplication with CD-HIT (58) (v4.8.1). Human and microbial rRNA reads were removed by alignment to SILVA rRNA references (59) (28S/18S/5.8S and 23S/16S/5S) using Bowtie2 (60) (v2.3.5.1, --local) and further filtered with SortMeRNA (61) (v4.2.0). Host-derived reads were then removed by alignment to the human reference genome (GRCh38.p13). The remaining non-host, non-rRNA reads were retained for microbial and functional analyses, whereas host-aligned reads were preserved for host genomic and transcriptomic analyses. Microbial abundance was quantified as reads per million (RPM), calculated as the number of reads assigned to a taxon divided by the total number of quality-controlled reads multiplied by 10⁶.

### Infectome identification

To systematically characterize the microbial landscape of each sample, preprocessed reads were analyzed using an in-house pipeline to define a curated clinical infectome. This framework integrates candidate screening, reference-based quantification, sequence validation, and background filtering to identify microorganisms with potential clinical relevance.

Candidate viral taxa were initially identified by nucleotide and translated similarity searches against the National Center for Biotechnology Information (NCBI) reference virus and non-redundant protein databases using BLASTn (62) (v2.15.0) and DIAMOND (63) blastx (v2.0.14), respectively. As all clinically relevant viral pathogens identified in this study corresponded to known species, reads were mapped to the closest representative reference genomes using Bowtie2 (--end-to-end, --very-fast) to estimate abundance and genome coverage. Reference-guided consensus sequences were reconstructed, and potential terminal misassemblies were assessed by BLASTn searches against the NCBI non-redundant nucleotide database (nt).

For cellular pathogens, reads were first mapped to an in-house reference database integrating GTDB (64) genomes for bacteria and archaea (release 214.1) with NCBI-derived fungal genomes selected from UFCG (65) references, together with clinically relevant *Pneumocystis* species, and assignments were initially summarized at the genus level. Reads assigned to candidate genera were extracted and re-mapped to species-level reference genomes downloaded from NCBI for candidate species within the corresponding genus (Table S5). Species identities were confirmed by reconstructing reference-guided consensus sequences from the second-pass alignments and validating them by BLASTn against the nt database, with additional manual verification for low-abundance or ambiguous cases.

To mitigate technical artifacts, two levels of background filtering were applied. First, an index-hopping filter removed taxon-specific reads that did not exceed 0.1% of the total reads assigned to the same taxon across all libraries within a sequencing lane. Second, sample type-specific thresholds derived from PC libraries were applied to remove taxa consistent with laboratory background. For each specimen type, taxa were discarded if their RPM did not exceed tenfold the maximum RPM observed for the same taxon in the corresponding PCs.

Following species-level sequence validation and technical background filtering, candidate detections were further curated to define the clinical infectome. A detection was retained if it met at least one of the following criteria: (i) it was supported by routine clinical testing results; (ii) it was detected across at least two parallel library strategies from the same sample; or (iii) in the absence of either clinical or cross-library support, its abundance exceeded 0.5 RPM. Unless otherwise specified, an infectome detection was considered recovered by an individual library strategy at an abundance of >0.5 RPM.

Taxon-specific conserved marker genes were used for phylogenetic validation and marker-gene coverage analyses of infectome species (Table S6). Marker sequences were reconstructed from mapped reads, aligned with MUSCLE (66), and used for maximum-likelihood phylogenetic inference with PhyML (67) 3.0 (v20120412). Only taxa with marker-gene coverage ≥ 80% were retained for phylogenetic reconstruction and visualization.

### Comparison with commonly used taxonomic classifiers

To evaluate the performance of commonly used computationally efficient taxonomic classifiers, Kraken2 (68) and MetaPhlAn4 (69) were applied to preprocessed non-host/non-rRNA reads. Kraken2 (v2.17.1) was run against the PlusPF 16 GB database released on 26 February 2026 using default parameters. MetaPhlAn4 (v4.2.4) was applied using the default marker-gene database to generate bacterial species-level profiles. The recovery of infectome taxa identified by our primary pipeline was evaluated by comparing species-level assignments from Kraken2 and MetaPhlAn4 with the curated clinical infectome benchmark.

### Functional profiling of resistance and virulence genes

Antibiotic resistance genes (ARGs) were identified by querying preprocessed non-host, non-rRNA reads against the Comprehensive Antibiotic Resistance Database (CARD) using the Resistance Gene Identifier (70) (v6.0.3) in bwt mode. To reduce spurious assignments, ARG hits were retained only if supported by more than 10 mapped reads, a mean mapping quality greater than 30, and mean coverage of at least 70%. To focus on interpretable sample-level resistome signals, downstream analyses were restricted to ARGs assigned to four canonical gene-mediated resistance mechanisms: antibiotic efflux, antibiotic inactivation, antibiotic target replacement, and antibiotic target protection. Filtered ARG hits were further mapped to antibiotic classes using CARD ontology annotations. For class-level comparisons across library types, a predefined panel of 14 representative antibiotic classes was selected to cover major antibiotic families as well as clinically important reserve classes, including glycylcyclines and oxazolidinones (Table S4).

Virulence factors were profiled by aligning preprocessed non-host, non-rRNA reads to the VFDB (71) Set A nucleotide database (exported 11 July 2025) using Bowtie2 (--end-to-end, --veryfast). VF hits were retained if supported by more than 10 mapped reads or at least 3 contiguous covered regions, each longer than 200 bp. To improve biological specificity, VF profiling was restricted to hits whose annotated source bacterium in VFDB matched a bacterial species detected in the infectome of the same sample.

For pathogen-level analyses of functional architecture, we focused on 5 bacterial pathogens that exhibited relatively high abundance (>4,000 reads in at least 1 library) and were detected in both DNAlib and RNAlib, including *Acinetobacter baumannii* and *Klebsiella pneumoniae*. Reads assigned to these pathogens were mapped to annotated reference genomes to quantify gene-level abundance. Resistance and virulence annotations were mapped to gene loci to construct rank–abundance profiles describing pathogen-specific functional architectures.

### Host transcriptomic profiling and gene-set scoring

To ensure robust host transcriptomic analyses, samples with fewer than 20,000 host-derived reads in any parallel library were excluded, yielding a final analysis subset of 71 samples. Preprocessed non-rRNA reads from the retained libraries were aligned to the human reference genome GRCh38.p13 using HISAT2 (72) (v2.1.0, --dta), and gene-level counts were quantified with featureCounts (73) based on the NCBI RefSeq GFF3 annotation (*Homo sapiens* Updated Annotation Release 109.20200228).

For cross-library comparisons of host gene-set signals, lowly expressed genes were filtered and gene counts were transformed to log₂-counts per million (log₂-CPM) using edgeR (v4.4.2) without additional between-sample normalization, such that normalization accounted only for library depth. Per-sample gene-set scores were then computed using singscore (74) (v1.19.2). To minimize specimen type–driven ranking effects, gene ranking and scoring were performed separately within each specimen type. Gene sets were obtained from MSigDB (75–77) and are listed in Table S7.

For analyses linking host transcriptional responses to microbial abundance in the BALF subset, DESeq2-normalized counts (78) (v1.46.0) were transformed using rlog, with size factors estimated by the poscounts method, and singscores were calculated from the resulting transformed expression values.

### Statistical analysis

All statistical analyses were performed in R (v4.4.2). Unless otherwise specified, tests were two-sided, and *P*-values were adjusted using the Benjamini–Hochberg procedure.

Within-sample comparisons across library types were assessed using paired Wilcoxon signed-rank tests, and unpaired comparisons using Wilcoxon rank-sum tests. Host genome recovery efficiency was evaluated using linear mixed-effects models with sample as a random intercept [Coverage ~ log10(host RPM + 1) × library type + (1|sample)], and library-specific slopes were estimated with emtrends. For host gene-set analyses, differences across infection-type groups were assessed using Wilcoxon rank-sum tests, and associations with microbial abundance were evaluated using linear regression models of the form singscore ~ bacterial abundance + viral abundance, with abundance defined as log₂-transformed summed RPM. *P*-values for slope comparisons and gene-set association analyses were corrected using the Benjamini–Hochberg method.

## Data Availability

Sequencing data generated in this study, with human reads removed, have been deposited in the NCBI Sequence Read Archive under BioProject accession number PRJNA1445946. Analysis code and processed data supporting the findings of this study are available on GitHub at https://github.com/LuoGengYan/mngs-library-comparison-code.

## SUPPLEMENTAL MATERIAL

Supplemental Figures and Tables msystems.00897-26-s0001.pdf Figures S1–S8 and Tables S1–S7.

ASM does not own the copyrights to Supplemental Material that may be linked to, or accessed through, an article. The authors have granted ASM a non-exclusive, world-wide license to publish the Supplemental Material files. Please contact the corresponding author directly for reuse.
