# Supplementary material for: Library strategies differentially shape microbial, functional, and host signals in clinical metagenomic sequencing
Source: mSystems. 2026 Sep 14;11(10):e00897-26. doi: 10.1128/msystems.00897-26 (PMC13712859; doi:10.1128/msystems.00897-26)
Supplement: Supplementary file 1 [file msystems.00897-26-s0001.pdf]

Supplemental Materials

Library strategies differentially shape microbial, functional and host signals in clinical metagenomic sequencing

Gengyan Luo[1,2], Jin Zhao[3], Lin-lin Xie[3], Yirong Li[3,4], Yang-zhi Rao[1,2], Yanni Sun[5], Xin Lu[6], John-Sebastian Eden[7,8], Liangjun Chen[#3,4], Mang Shi[#1,2,9]

[1]National Key Laboratory of Intelligent Tracking and Forecasting for Infectious Diseases, Zhongshan School of Medicine, Shenzhen Campus of Sun Yat-sen University, Sun Yat-sen University, Shenzhen, China.

[2]State Key Laboratory for Biocontrol, Zhongshan School of Medicine, Shenzhen Campus of Sun Yat-sen University, Sun Yat-sen University, Shenzhen, China.

[3]Department of Laboratory Medicine, Zhongnan Hospital of Wuhan University, Wuhan, China.

[4]Hubei Provincial Clinical Research Center for Molecular Diagnostics, Wuhan, China.

[5]Department of Electrical Engineering, City University of Hong Kong, Hong Kong, China.

[6]National Key Laboratory of Intelligent Tracking and Forecasting for Infectious Diseases, National Institute for Communicable Disease Control and Prevention, Chinese Center for Disease Control and Prevention, Beijing, China.

[7]Sydney Institute for Infectious Diseases, The University of Sydney, Sydney, New South Wales, Australia.

[8]Centre for Virus Research, Westmead Institute for Medical Research, Sydney, New South Wales, Australia.

[9]Institute of Parasitic Disease Control and Prevention, Center for Disease Control and Prevention of Guangdong Province, Guangzhou, China.

Running Head: Library Strategies Shape Clinical Infectome Signals

#Corresponding authors: Mang Shi (shim23@mail.sysu.edu.cn), Liangjun Chen (chenliangjun@whu.edu.cn)

Supplemental Figures

Figure S1. Viral phylogenetic analysis of the clinical infectome using marker-gene trees.

Figure S2. Phylogenetic analysis of cellular microbes in the clinical infectome using marker-gene trees.

Figure S3. Recovery of the clinical infectome by Kraken2 and MetaPhlAn4.

Figure S4. Sample-type-stratified performance of mNGS library strategies.

Figure S5. Threshold-dependent detection and abundance patterns across finer taxonomic ranks.

Figure S6. Representative species-level examples illustrating library-type–specific infectome abundance patterns.

Figure S7. ARG-associated antibiotic class annotations in non-BALF specimen types.

Figure S8. Host read metrics and infection-type–stratified host gene-set activities.

Supplemental Tables

Table S1. Sample metadata, mNGS-identified infectome species, and corresponding clinically reported pathogens for each sample.

Table S2. Comparison of cost and efficiency across the three mNGS library strategies.

Table S3. Species-level detection frequency and positivity rates of the infectome across library types.

Table S4. Predefined panel of 14 representative antibiotic classes used for resistome profiling.

Table S5. Reference genomes used for mapping analyses in species identification.

Table S6. Conserved marker genes used for taxonomic identification and phylogenetic analysis.

Table S7. Host gene sets and functional modules used for transcriptomic profiling.

Supplemental Figures

Figure S1

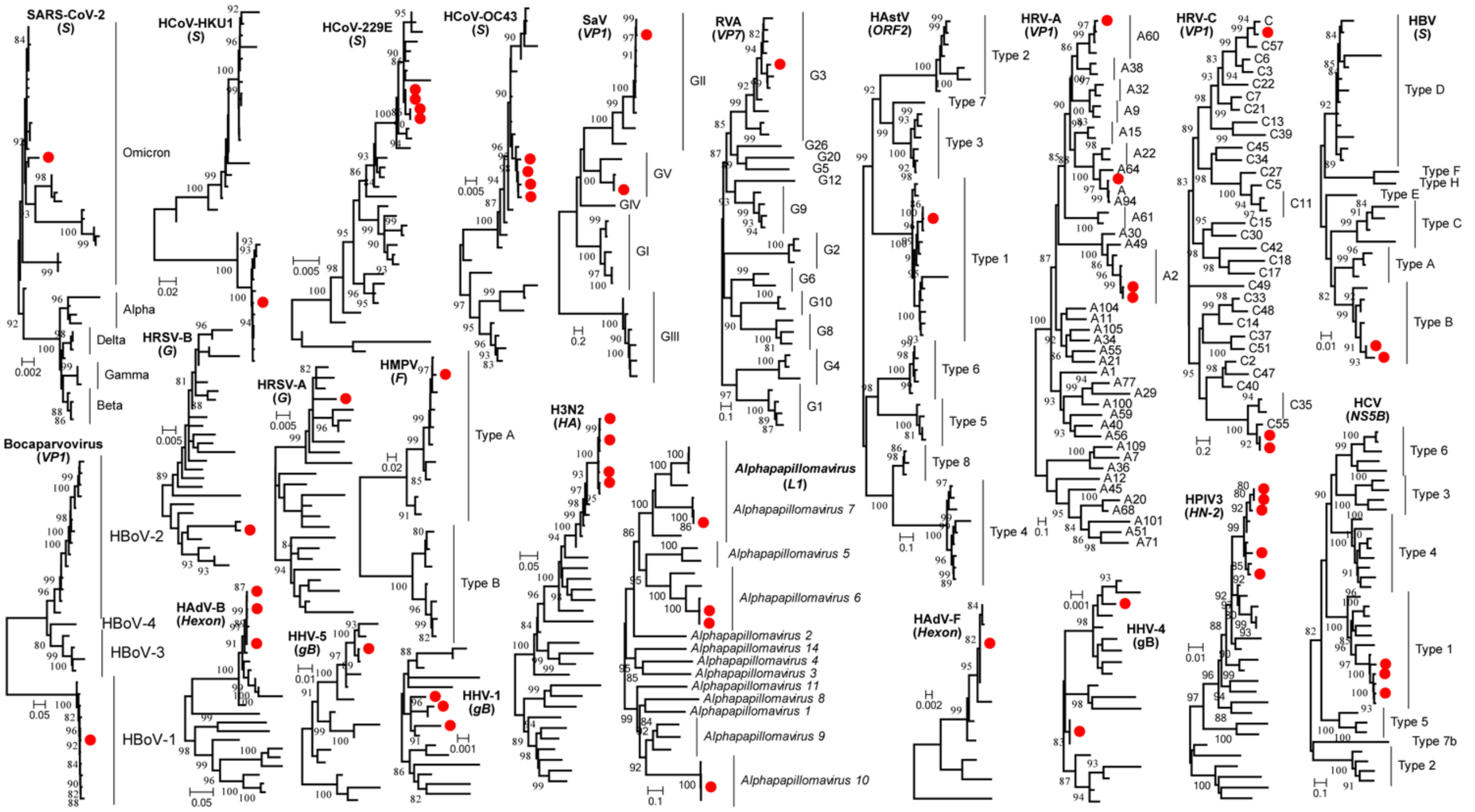

Figure S1. Viral phylogenetic analysis of the clinical infectome using marker-gene trees. Maximum-likelihood (ML) phylogenetic trees were constructed for viral taxa detected in the clinical infectome. Each tree is labeled with the corresponding viral group and the phylogenetic marker gene used for tree construction (shown in parentheses). Red dots indicate sequences recovered from the clinical cohort. Branch support values are shown on branches, with values <80 not shown. The scale bar represents the number of nucleotide substitutions per site.

Figure S2

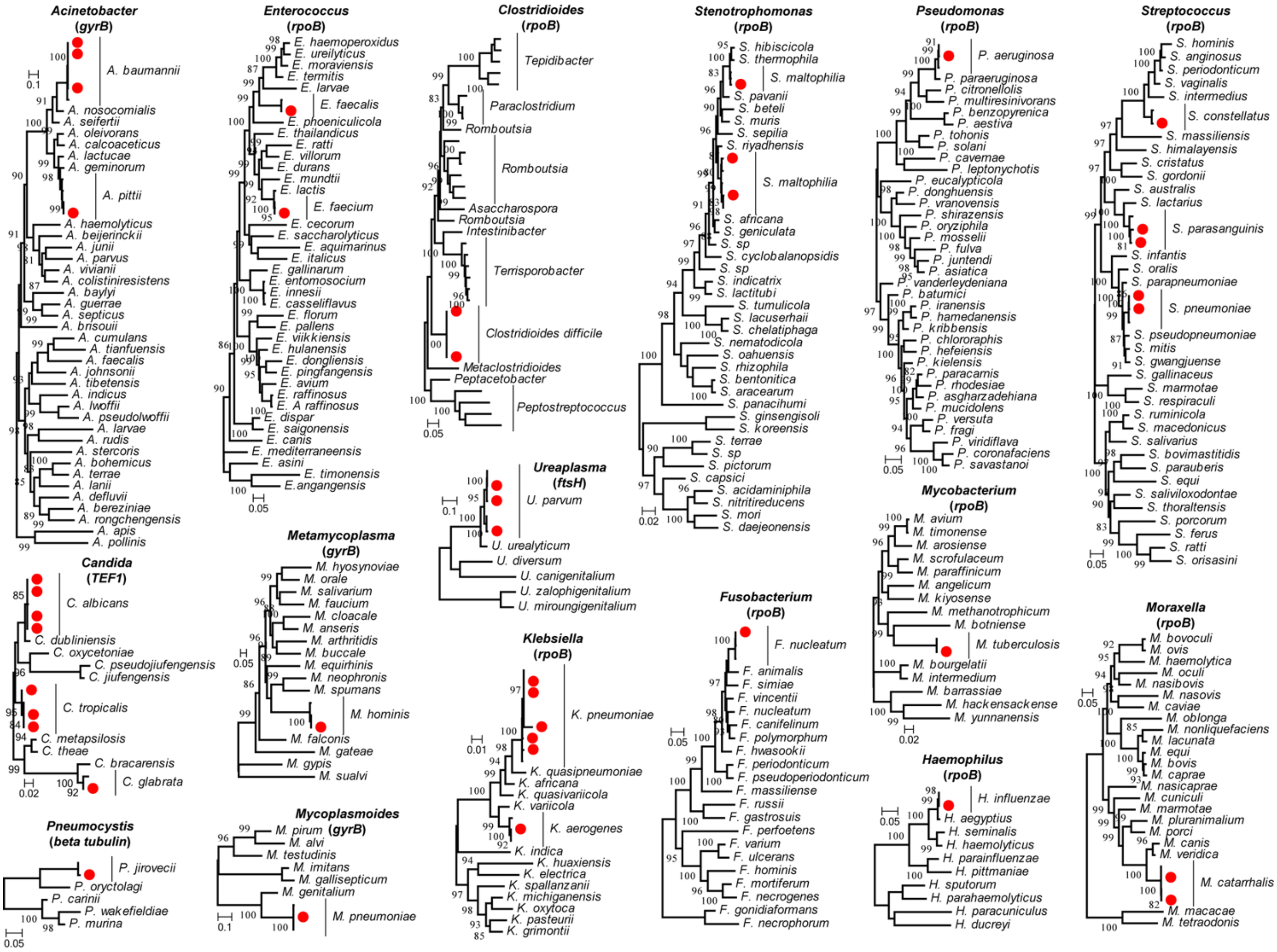

Figure S2. Phylogenetic analysis of cellular microbes in the clinical infectome using marker-gene trees.

Maximum-likelihood (ML) phylogenetic trees were constructed for cellular microbes (bacteria, fungi, and other cellular pathogens, including *Mycoplasmoides*) detected in the clinical infectome. Each tree is labeled with the corresponding taxonomic group and the phylogenetic marker gene used for tree construction (shown in parentheses). Red dots indicate sequences recovered from the clinical cohort. Branch support values are shown on branches, with values <80 not shown. The scale bar represents the number of nucleotide substitutions per site.

Figure S3

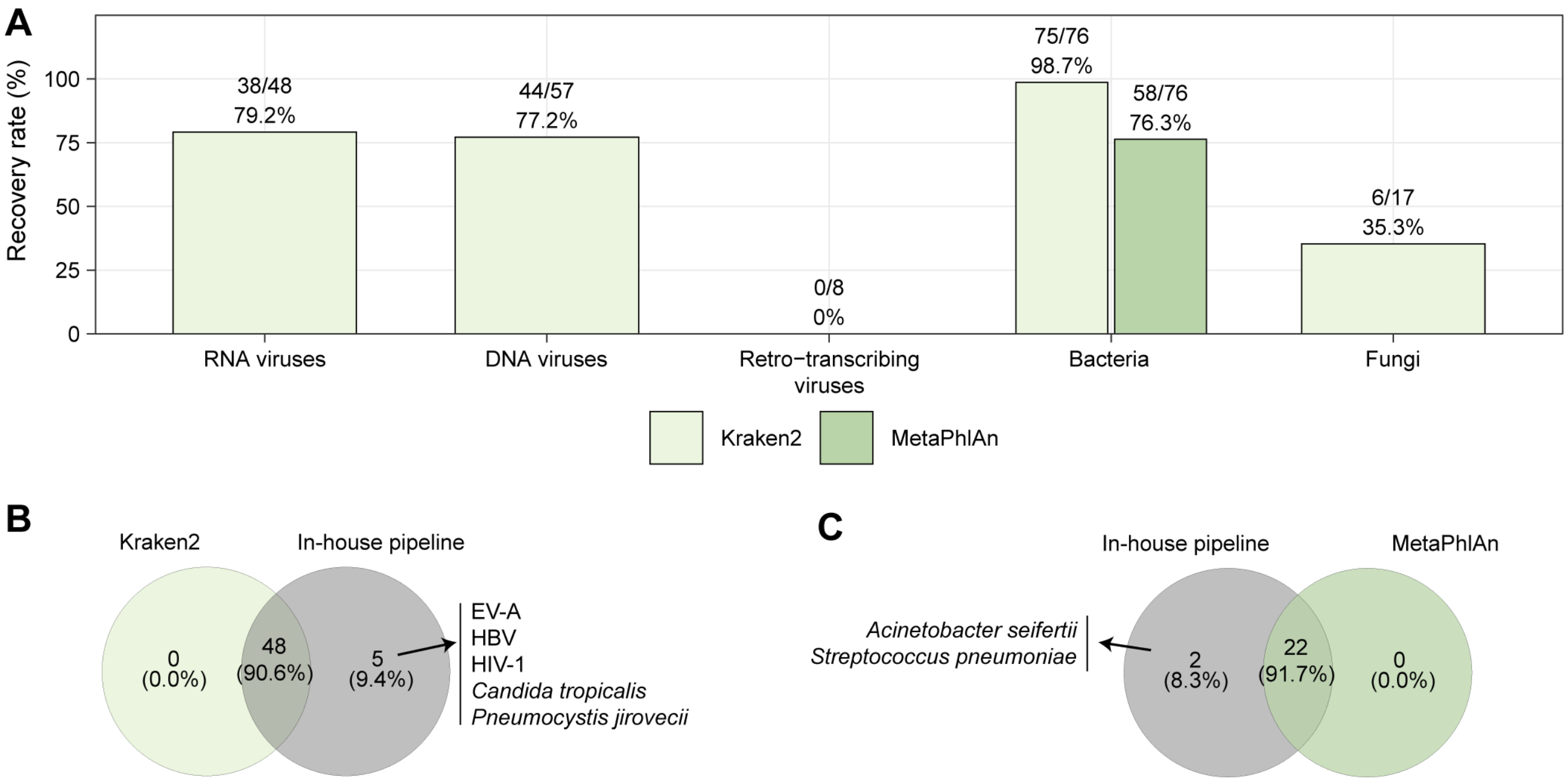

Figure S3. Recovery of the clinical infectome by Kraken2 and MetaPhlAn4.

(A) Recovery rates of infectome by Kraken2 across microbial categories and by MetaPhlAn4 for bacterial detections. Fractions above the bars indicate the number of detections recovered by each classifier relative to the total number identified by the in-house pipeline.

(B) Overlap between the 53 distinct infectome species identified by the in-house pipeline and those recovered by Kraken2. Species not recovered by Kraken2 are indicated.

(C) Overlap between the 24 distinct bacterial infectome species identified by the in-house pipeline and those recovered by MetaPhlAn4. Species not recovered by MetaPhlAn4 are indicated.

Figure S4

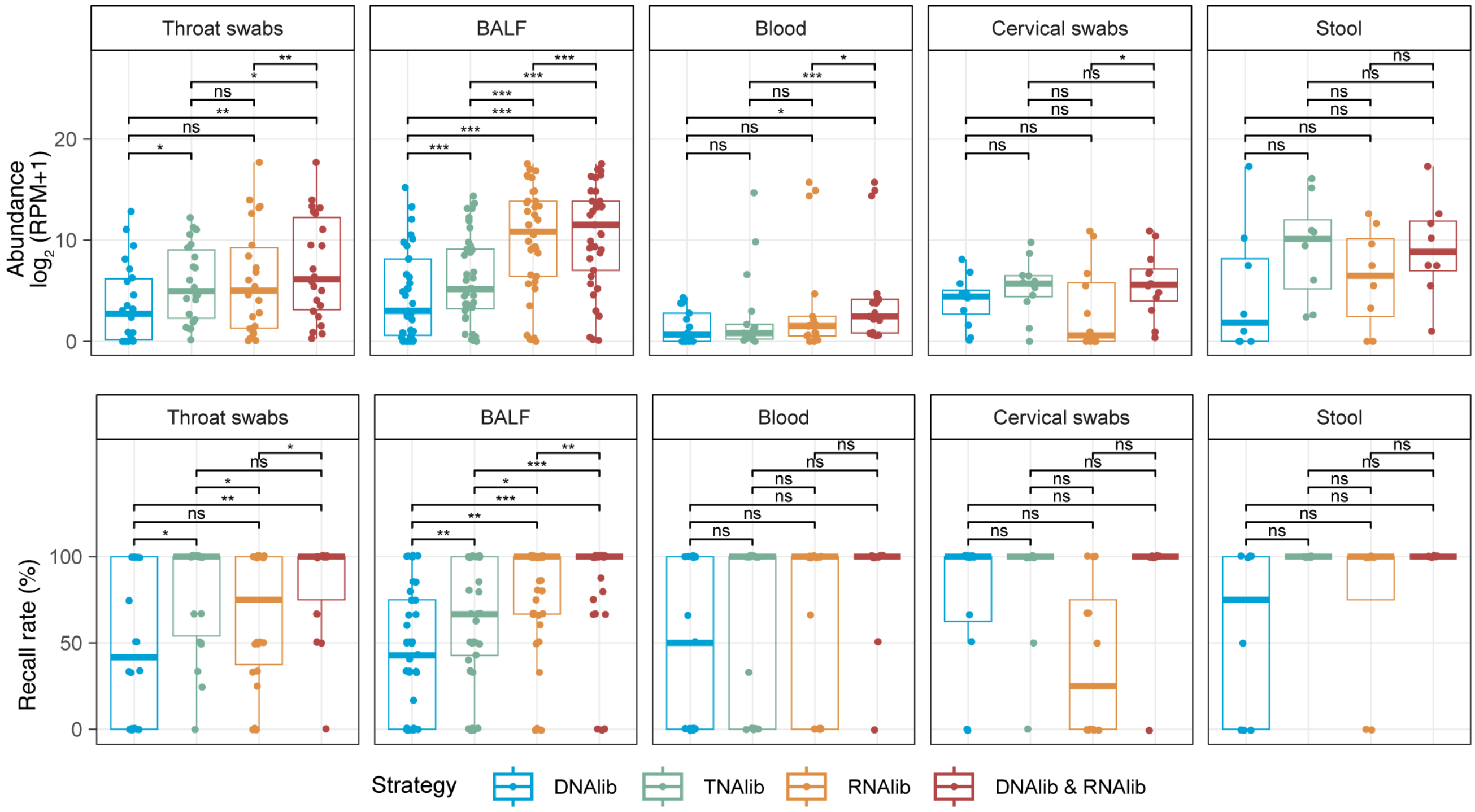

Figure S4. Sample-type-stratified performance of mNGS library strategies.

(A) Sample-level infectome abundance across DNAlib, TNAlib, RNAlib, and the combined DNAlib & RNAlib strategy, stratified by specimen type. Abundance is shown as $\log_2(\text{RPM} + 1)$, where RPM represents the summed abundance of all infectome species detected in each specimen.

(B) Sample-level infectome recall rates across the same four strategies, stratified by specimen type. For all boxplots, center lines indicate medians and boxes represent interquartile ranges (IQRs). Analyses included 96 clinical specimens, with each point representing one specimen. Statistical significance was assessed using paired Wilcoxon signed-rank tests with Benjamini–Hochberg correction for multiple comparisons and is indicated as ns, not significant; $*P < 0.05$; $**P < 0.01$; and $***P < 0.001$.

Figure S5

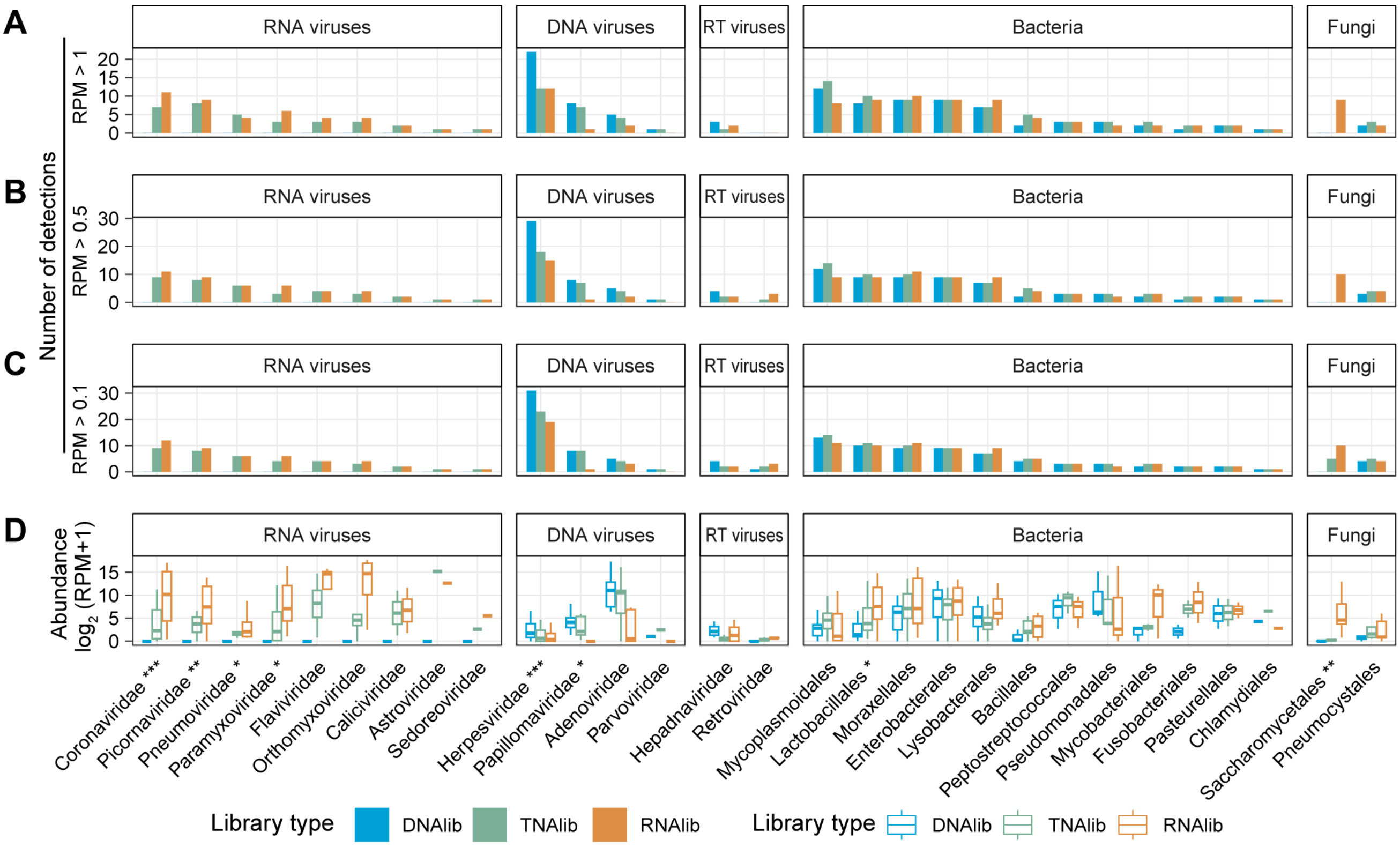

Figure S5. Threshold-dependent detection and abundance patterns across finer taxonomic ranks.

(A–C) Detection counts of the infectome across taxonomic ranks (viral families and bacterial/fungal orders) at RPM cutoffs of >1 (A), >0.5 (B), and >0.1 (C). Bar plots show the number of species detections for each library type (DNAlib, TNAlib, and RNAlib); RT viruses denote retro-transcribing viruses.

(D) Taxon-level infectome abundance across microbial categories. Boxplots summarize abundance across library types, expressed as $\log_2(\text{RPM} + 1)$, where each point represents the abundance of an individual detected taxon. Asterisks next to taxon labels indicate significant DNAlib–RNAlib abundance differences for that taxon (paired Wilcoxon signed-rank tests with Benjamini–Hochberg correction), annotated as $*P < 0.05$, $**P < 0.01$, and $***P < 0.001$.

Figure S6

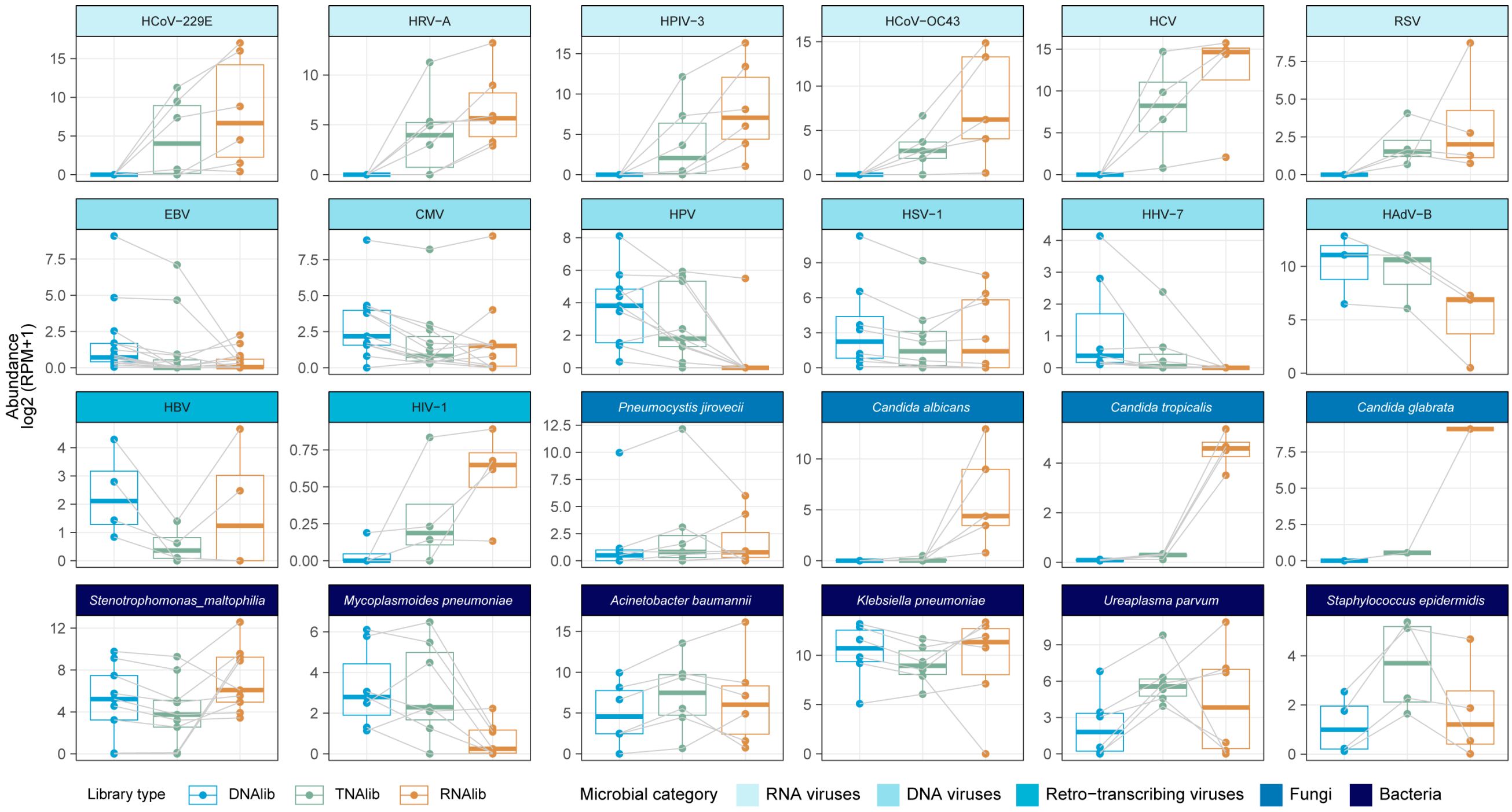

Figure S6. Representative species-level examples illustrating library-type–specific infectome abundance patterns.

Faceted boxplots depict the abundance of selected representative infectome taxa across three library preparation strategies (DNAlib, TNAlib, and RNAlib), expressed as $\log_2$(RPM + 1). In boxplots, center lines indicate medians and boxes indicate interquartile ranges (IQRs). Points represent individual detections, and gray lines connect paired measurements from the same clinical specimen across library types.

Figure S7

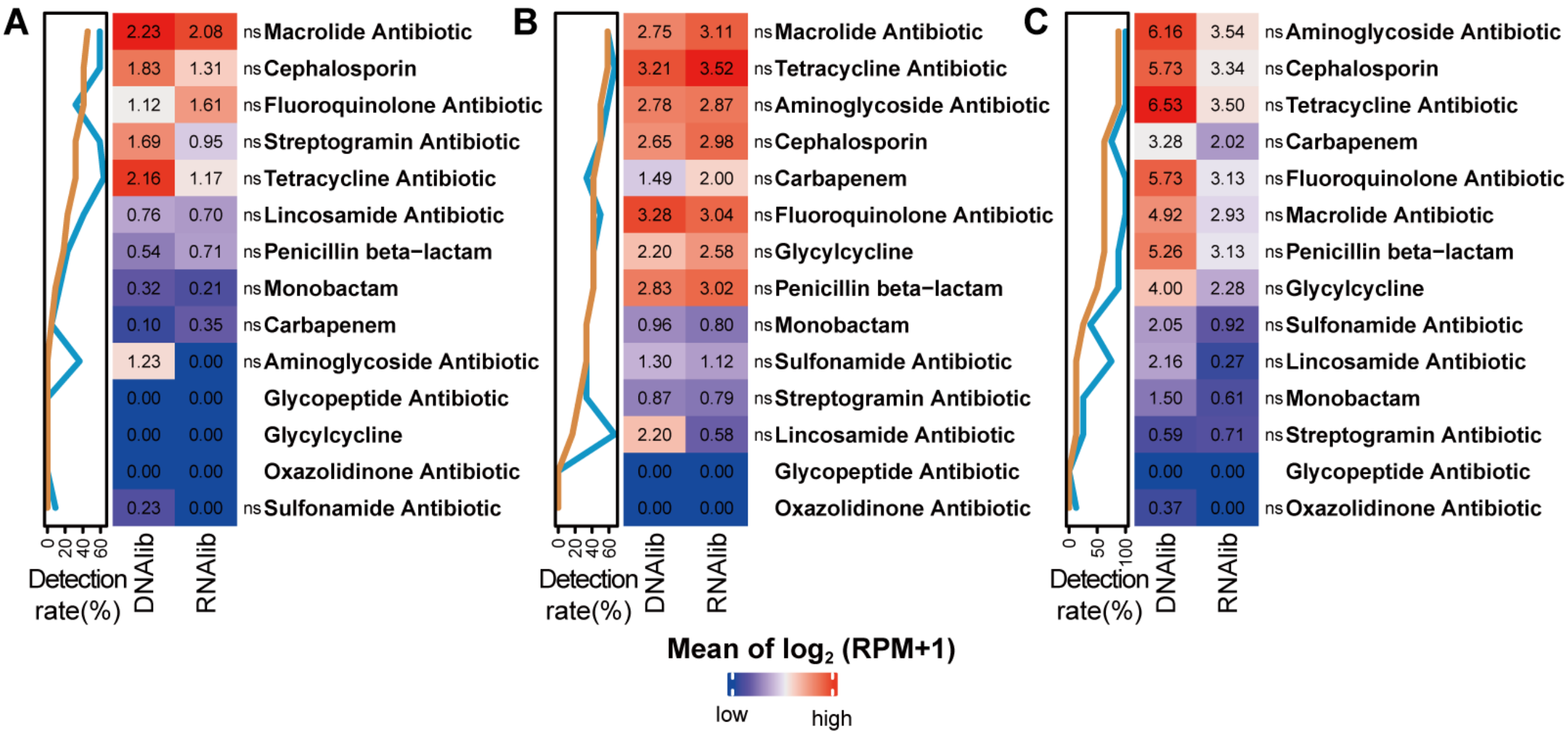

Figure S7. ARG-associated antibiotic class annotations in non-BALF specimen types.

Comparison of ARG-associated antibiotic class annotations between DNAlib and RNAlib across three non-BALF specimen types: (A) throat swabs, (B) cervical swabs, and (C) stool. Analyses are based on 14 representative antibiotic classes (see Table S4 and Materials and Methods for details). For each specimen type, line plots show the detection rate of each antibiotic class, and the heatmap displays the mean abundance of detected ARG annotations associated with each class, expressed as mean $\log_2$(RPM+1). Significance annotations next to antibiotic class labels indicate DNAlib–RNAlib differences for each class assessed using paired Wilcoxon signed-rank tests with Benjamini–Hochberg (BH) correction for multiple comparisons: ns (not significant); *$P < 0.05$; **$P < 0.01$; ***$P < 0.001$.

Figure S8

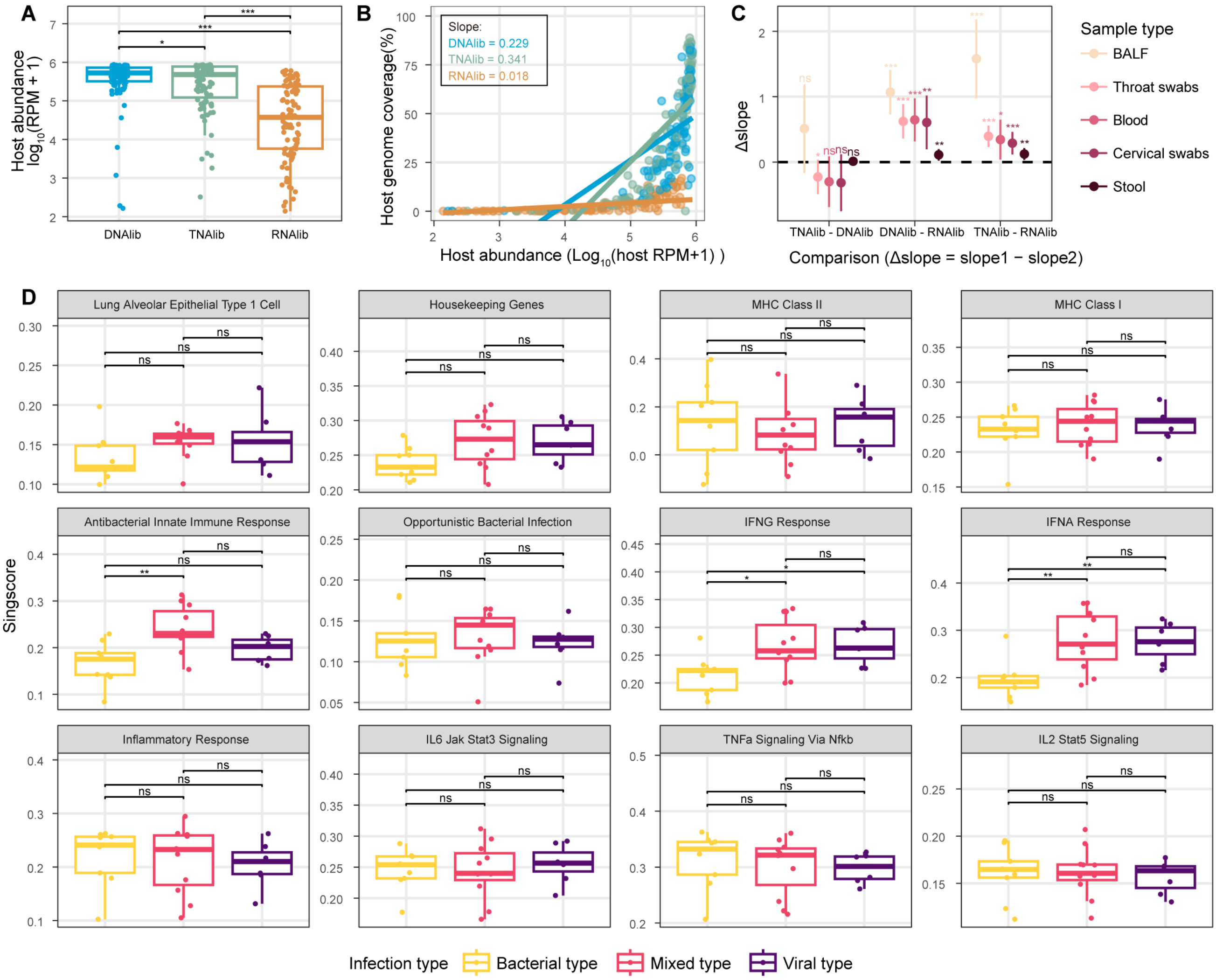

Figure S8. Host read metrics and infection-type–stratified host gene-set activities.

(A) Host read abundance across library preparation strategies, shown as $\log_{10}(RPM + 1)$.

(B) Relationship between host read abundance and host genome coverage. Scatter plots show host genome coverage versus host read abundance (expressed as $\log_{10}(RPM + 1)$), with points colored by library type. Solid lines indicate fitted relationships from a linear mixed-effects model with sample as a random intercept.

(C) Variations in coverage–host abundance scaling across specimen types. Forest plots show model-based slope differences (Δslope) for the indicated library-type comparisons within each specimen type; points indicate estimates and horizontal error bars denote 95% confidence intervals.

(D) Host gene-set activity in BALF samples with sufficient host read depth (n = 32), stratified by infection type. Faceted boxplots show singscore distributions across viral, mixed, and bacterial infection types for representative gene sets at an RPM cutoff of >10.

In boxplots (A and D), center lines indicate medians and boxes indicate interquartile ranges (IQRs); points represent individual samples. Statistical significance was assessed using paired Wilcoxon signed-rank tests for (A), model-based slope contrasts for (C), and Wilcoxon rank-sum tests for (D), with Benjamini–Hochberg (BH) correction for multiple comparisons and is annotated as ns (not significant), *$P < 0.05$, **$P < 0.01$, and ***$P < 0.001$.

Supplemental Tables

Table S1. Sample metadata, mNGS-identified infectome species, and corresponding clinically reported pathogens for each sample.

Sample ID	Sample type	Infectome identified by mNGS	Infectome described in clinical report
G56	BALF	*Acinetobacter baumannii, Enterococcus faecium, Klebsiella aerogenes, Candida glabrata*, EV-A	*Canidia albicans*
BF02	BALF	*Acinetobacter baumannii, Klebsiella pneumoniae, Morganella morganii, Stenotrophomonas maltophilia,* CMV, HSV-1, HPIV-3	*Acinetobacter baumannii, Klebsiella pneumoniae, Morganella morganii*, CMV, HSV-1, HPIV-3
G11	BALF	*Acinetobacter baumannii, Enterococcus faecalis, Klebsiella aerogenes, Klebsiella pneumoniae, Pseudomonas aeruginosa, Stenotrophomonas maltophilia,* HHV-7, HRV-A	*Acinetobacter baumannii, Pseudomonas aeruginosa*
G16	BALF	*Acinetobacter baumannii,* EBV, HSV-1	Not assessed
G17	BALF	*Acinetobacter baumannii*, CMV,HSV-1,HAdV F,HRV-A	Not assessed
G31410	BALF	*Acinetobacter baumannii, Candida albicans*	*Acinetobacter baumannii*
31809	BALF	*Acinetobacter seifertii, Enterococcus faecalis, Klebsiella pneumoniae, Candida albicans, Stenotrophomonas maltophilia,*EBV,HSV-1	*Klebsiella pneumoniae, Stenotrophomonas maltophilia*
BF07	BALF	*Acinetobacter pittii, Enterococcus faecalis, Candida albicans, Stenotrophomonas maltophilia*, CMV, HSV-1, HCoV-229E, HPIV-3	*Acinetobacter pittii, Enterococcus faecalis, Candida albicans, Stenotrophomonas maltophilia*, CMV, HSV-1, HCoV-229E, HPIV-3
FBZJ02	BALF	*Enterococcus faecium*, HCoV-HKU1, *Pneumocystis jirovecii*	*Aspergillus flavus, Aspergillus oryzae, Pneumocystis jirovecii*, HCoV-HKU1
FBZJ06	BALF	*Enterococcus faecium*, HRV-C, *Pneumocystis jirovecii*	*Pneumocystis jirovecii*
ZJ02	BALF	*Haemophilus influenzae, Candida albicans*, IAV(H3N2)	*Haemophilus influenzae, Canidia albicans,Aspergillus fumigatus*, IAV(H3N2)
G14	BALF	*Klebsiella pneumoniae, Stenotrophomonas maltophilia*, EBV, HSV-1	Not assessed
G30055	BALF	*Klebsiella pneumoniae,Staphylococcus aureus*	*Klebsiella pneumoniae*

G31608	BALF	*Klebsiella pneumoniae, Staphylococcus epidermidis,* HSV-1, HCoV-OC43	*Klebsiella pneumoniae*
24-TB02	BALF	*Morganella morganii, Mycobacterium tuberculosis, Stenotrophomonas maltophilia*	*Mycobacterium tuberculosis*
24-TB01	BALF	*Mycobacterium tuberculosis*	*Mycobacterium tuberculosis*
24-TB03	BALF	*Mycobacterium tuberculosis, Stenotrophomonas maltophilia*	*Mycobacterium tuberculosis*
BF05	BALF	*Pseudomonas aeruginosa*, HPIV-3	*Pseudomonas aeruginosa, Canidia albicans*, HPIV-3
G30327	BALF	*Candida albicans,Stenotrophomonas maltophilia*	*Candida tropicalis*
G67	BALF	*Candida tropicalis, Staphylococcus epidermidis, Pneumocystis jirovecii,* CMV, EBV	*Candida tropicalis*
G79	BALF	*Candida tropicalis*, EBV, HRV-A	*Candida tropicalis*
G31819	BALF	*Candida tropicalis,Staphylococcus aureus,Streptococcus pneumoniae*	*Aspergillus niger*
G44	BALF	*Candida tropicalis,Fusobacterium nucleatum*	Not assessed
31821	BALF	*Stenotrophomonas maltophilia,Staphylococcus epidermidis*,HCoV-OC43	*Stenotrophomonas maltophilia*
BF03	BALF	*Mycoplasmoides pneumoniae*	*Mycoplasmoides pneumoniae*,HCoV-229E
BF08	BALF	*Staphylococcus epidermidis, Streptococcus parasanguinis*, CMV,EBV	*Streptococcus parasanguinis,Aspergillus terreus*, CMV, HCoV-229E
BF06	BALF	*Pneumocystis jirovecii*, CMV, HCoV-229E, HRV-C, RSV, HPIV-3	*Enterococcus faecium, Pneumocystis jirovecii, Aspergillus fumigatus,* CMV, HRV-C, RSV
G05	BALF	*Pneumocystis jirovecii,* EBV, SARS-CoV-2	*Pneumocystis jirovecii,* CMV, SARS-CoV-2, HRV-C
G35	BALF	EBV, *Streptococcus parasanguinis*	Not assessed
ZJ07	BALF	HRV-C, *Pneumocystis jirovecii*	*Aspergillus flavus, Aspergillus oryzae, Pneumocystis jirovecii*, HRV-C
G08	BALF	HHV-7, *Pneumocystis jirovecii*	*Chlamydia psittaci*, HHV-7, *Pneumocystis jirovecii*
G32254	BALF	HSV-1	Not assessed
BF04	BALF	HCoV-229E,HPIV-3	*Streptococcus pneumoniae,* CMV, HPIV-3, HCoV-229E
BF01	BALF	HCoV-229E	*Pneumocystis jirovecii,*HCoV-229E

G47	BALF	HCoV-OC43	*Candida parapsilosis*
G76	BALF	HCoV-OC43	Not assessed
G31	BALF	*Fusobacterium nucleatum, Streptococcus constellatus*	Not assessed
B25	Blood/Whole blood	CMV	*Morganella morganii*
M01	Blood/Whole blood	CMV	CMV
M02	Blood/Whole blood	CMV	CMV
M03	Blood/Whole blood	CMV	CMV
M04	Blood/Whole blood	CMV	CMV
H05	Blood/Serum	HIV-1	HIV-1
H09	Blood/Serum	HBV,HIV-1	HIV-1
B26	Blood/Whole blood	EBV	*Pseudomonas aeruginosa*
E02	Blood/Whole blood	EBV,HBV,HIV-1	EBV
E03	Blood/Whole blood	EBV	EBV
24-HCV01	Blood/Serum	HCV	HCV
24-HCV02	Blood/Serum	HCV	HCV
C04	Blood/Serum	HCV	HCV
C05	Blood/Serum	HCV	HCV
B02	Blood/Whole blood	HBV	HBV
B09	Blood/Whole blood	HBV	*Staphylococcus epidermidis*
H03	Blood/Serum	HIV-1	HIV-1
Y01_20231209	Cervical swabs	*Chlamydia trachomatis*	*Chlamydia*
Z03	Cervical swabs	*Ureaplasma parvum*, HPV	*Ureaplasma parvum*
Z06	Cervical swabs	*Ureaplasma parvum*	*Ureaplasma parvum*
Z07	Cervical swabs	*Ureaplasma parvum*	*Ureaplasma parvum*
P04	Cervical swabs	*Ureaplasma parvum, Metamycoplasma hominis, HPV*	HPV
P06	Cervical swabs	*Ureaplasma parvum, Metamycoplasma hominis, HPV*	HPV

P09	Cervical swabs	*Ureaplasma parvum,HPV*	HPV
HPV02	Cervical swabs	*HPV*	HPV
P03	Cervical swabs	*HPV*	HPV
P02	Cervical swabs	*HPV*	HPV
P10	Cervical swabs	*HPV*	HPV
HPV04	Cervical swabs	*HPV*	HPV
F98	Stool	HAdV F	Not assessed
F91	Stool	HAstV	Not assessed
F09	Stool	RVA	Not assessed
F100	Stool	HBoV	Not assessed
F01	Stool	SaV, *Clostridioides difficile*	Not assessed
F95	Stool	SaV	Not assessed
F92	Stool	*Clostridioides difficile*	Not assessed
F94	Stool	*Clostridioides difficile*	Not assessed
M123	Throat swabs	*Haemophilus influenzae, Mycoplasmoides pneumoniae*	*Mycoplasmoides pneumoniae*, HAdV-B
Y10	Throat swabs	*Pseudomonas aeruginosa*, EBV, HHV-7	Not assessed
M031	Throat swabs	*Mycoplasmoides pneumoniae*, HAdV B	*Mycoplasmoides pneumoniae*, HAdV-B
M171	Throat swabs	*Mycoplasmoides pneumoniae*,HAdV B	*Mycoplasmoides pneumoniae*, HAdV-B
MP04	Throat swabs	*Mycoplasmoides pneumoniae*, EBV, HHV-7	*Mycoplasmoides pneumoniae*
MP06	Throat swabs	*Mycoplasmoides pneumoniae*,HCoV-229E,HPIV-3	*Mycoplasmoides pneumoniae*
MP08	Throat swabs	*Mycoplasmoides pneumoniae*, EBV, HHV-7, HMPV	*Mycoplasmoides pneumoniae*
Y09	Throat swabs	*Moraxella catarrhalis*, HRV-A	Not assessed
M068	Throat swabs	EBV, HCoV-229E,RSV	RSV
Y01	Throat swabs	EBV, HHV-7, HRV-A	Not assessed
M158	Throat swabs	HHV-7, RSV	RSV
Y07	Throat swabs	HHV-7, HRV-A	Not assessed
Y11	Throat swabs	HHV-7,HCoV-OC43	Not assessed
M055	Throat swabs	HAdV-B	*Mycoplasmoides pneumoniae*, HAdV-B
M073	Throat swabs	HMPV	HMPV
M130	Throat swabs	HMPV	HMPV
M127	Throat swabs	RSV	RSV
L02	Throat swabs	IAV(H3N2)	IAV(H3N2)

L04	Throat swabs	IAV(H3N2), *Streptococcus pneumoniae*	IAV(H3N2)
M086	Throat swabs	IAV(H3N2)	HMPV, IAV(H3N2)
L03	Throat swabs	*Moraxella catarrhalis*	IAV(H3N2)
MP07	Throat swabs	*Acinetobacter pittii*	*Mycoplasmoides pneumoniae*

Table S2. Comparison of cost and efficiency across the three mNGS library strategies.

Table S2. Comparison of cost and efficiency across the three mNGS library strategies.

Item	DNAlib	TNAlib	RNAlib
Major workflow steps	DNA library construction	cDNA synthesis + DNA library construction	DNase treatment + rRNA depletion + cDNA synthesis + DNA library construction
Approximate reagent cost per sample	US$22.10	US$29.50	US$60.40
Hands-on time	0.6 h	1 h	1.6 h
Library preparation time	1.8 h	3.7 h	6.8 h

Values represent approximate workflow-level estimates for processing a single sample under the laboratory implementation used in this study.

Major workflow steps indicate the principal library-preparation modules performed after total nucleic acid extraction.

Reagent costs refer to library preparation-related reagents per sample and exclude nucleic acid extraction, sequencing, labor, equipment depreciation, shipping, taxes, and bioinformatic analysis.

Hands-on time refers to the cumulative time requiring active operator manipulation for processing a single sample.

Library preparation time refers to the approximate elapsed time from extracted total nucleic acid input to a sequencing-ready library for a single sample, including active manipulation, incubation, reaction, PCR, and purification steps.

Table S3. Species-level detection frequency and positivity rates of the infectome across library types.

Infectome	Microbial group	Number of DNAlib-positive samples	Number of TNAlib-positive samples	Number of RNAlib-positive samples	Number of DNAlib & RNAlib-positive samples	Overall positivity rate
HAstV	RNA viruses	0	1	1	1	1.04%
HCoV-229E	RNA viruses	0	4	5	5	6.25%
HCoV-HKU1	RNA viruses	0	0	1	1	1.04%
HCoV-OC43	RNA viruses	0	4	4	4	5.21%
EV-A	RNA viruses	0	1	0	0	1.04%
IAV(H3N2)	RNA viruses	0	3	4	4	4.17%
HCV	RNA viruses	0	4	4	4	4.17%
HMPV	RNA viruses	0	2	2	2	3.13%
HPIV3	RNA viruses	0	3	6	6	6.25%
HRV-A	RNA viruses	0	4	6	6	6.25%
HRV-C	RNA viruses	0	3	3	3	3.13%
RSV	RNA viruses	0	4	4	4	1.04%
RVA	RNA viruses	0	1	1	1	4.17%
SARS CoV 2	RNA viruses	0	1	1	1	1.04%
SaV	RNA viruses	0	2	2	2	2.08%
HAdV-B	DNA viruses	3	3	2	3	3.13%
HAdV-F	DNA viruses	2	1	0	2	2.08%

HBoV	DNA viruses	1	1	0	1	1.04%
HSV-1	DNA viruses	6	5	4	6	8.33%
EBV	DNA viruses	11	4	4	13	16.67%
CMV	DNA viruses	10	7	7	11	11.46%
HHV-7	DNA viruses	2	2	0	2	11.46%
HPV	DNA viruses	8	7	1	8	9.38%
HBV	Retro-transcribing viruses	4	2	2	4	4.17%
HIV-1	Retro-transcribing viruses	0	1	3	3	4.17%
Acinetobacter baumannii	Bacteria	5	6	6	6	6.25%
Acinetobacter pittii	Bacteria	2	2	2	2	2.08%
Acinetobacter seifertii	Bacteria	0	0	1	1	1.04%
Chlamydia trachomatis	Bacteria	1	1	1	1	1.04%
Clostridioides difficile	Bacteria	3	3	3	3	3.13%
Enterococcus faecium	Bacteria	2	3	2	3	3.13%
Enterococcus faecalis	Bacteria	2	2	3	3	3.13%
Fusobacterium nucleatum	Bacteria	1	2	2	2	2.08%
Haemophilus influenzae	Bacteria	2	2	2	2	2.08%
Klebsiella aerogenes	Bacteria	2	2	2	2	2.08%
Klebsiella pneumoniae	Bacteria	6	6	5	6	6.25%
Metamycoplasma hominis	Bacteria	2	2	2	2	2.08%
Moraxella catarrhalis	Bacteria	2	2	2	2	2.08%

Morganella morganii	Bacteria	1	1	2	2	2.08%
Mycobacterium tuberculosis	Bacteria	2	3	3	3	3.13%
Mycoplasmoides pneumoniae	Bacteria	7	6	3	7	7.29%
Pseudomonas aeruginosa	Bacteria	3	3	2	3	3.13%
Staphylococcus aureus	Bacteria	0	1	2	2	2.08%
Staphylococcus epidermidis	Bacteria	2	4	2	3	4.17%
Stenotrophomonas maltophilia	Bacteria	7	7	9	9	9.38%
Streptococcus constellatus	Bacteria	1	1	1	1	1.04%
Streptococcus parasanguinis	Bacteria	2	2	2	2	2.08%
Streptococcus pneumoniae	Bacteria	2	2	1	2	2.08%
Ureaplasma parvum	Bacteria	3	6	4	6	6.25%
Candida albicans	Fungi	0	0	5	5	5.21%
Candida glabrata	Fungi	0	0	1	1	1.04%
Candida tropicalis	Fungi	0	0	4	4	4.17%
Pneumocystis jirovecii	Fungi	3	4	4	5	7.29%

Table S4. Predefined panel of 14 representative antibiotic classes used for resistome profiling.

**Antibiotic Class (CARD Ontology)**	**Clinical Spectrum & Primary Role**	**Representative Examples**
Penicillin beta-lactam	Broad-spectrum empirical & targeted therapy	Amoxicillin, Piperacillin-tazobactam
Cephalosporin	Broad-spectrum empirical & targeted therapy (generation-dependent)	Ceftriaxone, Ceftaroline
Macrolide antibiotic	Empirical therapy covering respiratory & atypical pathogens	Azithromycin, Clarithromycin
Fluoroquinolone antibiotic	Broad-spectrum empirical therapy (including atypicals)	Ciprofloxacin, Levofloxacin
Aminoglycoside antibiotic	Targeted therapy for aerobic Gram-negative infections	Gentamicin, Amikacin
Monobactam	Targeted therapy for aerobic Gram-negative infections (e.g., Pseudomonas)	Aztreonam
Carbapenem	Broad-spectrum salvage therapy for severe MDR Gram-negative infections	Meropenem, Imipenem
Lincosamide antibiotic	Targeted therapy for Gram-positive & anaerobic infections	Clindamycin
Glycopeptide antibiotic	Targeted salvage therapy for MDR Gram-positive infections (e.g., MRSA)	Vancomycin, Teicoplanin
Oxazolidinone antibiotic	Targeted salvage therapy for MDR Gram-positive infections (e.g., VRE, MRSA)	Linezolid
Streptogramin antibiotic	Targeted salvage therapy for MDR Gram-positive infections (e.g., VRE)	Quinupristin-dalfopristin
Tetracycline antibiotic	Broad-spectrum therapy for atypical & intracellular pathogens	Doxycycline, Minocycline
Glycylcycline	Broad-spectrum salvage therapy for complex MDR infections	Tigecycline
Sulfonamide antibiotic	Targeted therapy for specific opportunistic (e.g., Pneumocystis) & urinary infections	Sulfamethoxazole-trimethoprim

Table S5. Reference genomes used for mapping analyses in species identification.

Infectome taxon	Reference genome accession(s)
HAstV	PQ496891.1
HCoV-229E	NC_002645.1
HCoV-HKU1	NC_006577.2
HCoV-OC43	NC_006213.1
EV-A	OR500230.1
IAV(H3N2)	GCF_000865085.1
HCV	GU451221.1, KC844052.1, KJ678771.1
HMPV	NC_039199.1
HPIV3	NC_075446.1
HRV-A	MH899591.1, MN749152.1, MW587076.1, MZ835588.1, ON729329.1
HRV-C	LC720415.1, NC_038878.1
RSV	NC_001803.1, NC_001781.1
RVA	GCA_002639295.1
SARS CoV 2	NC_045512.2
SaV	LC788819.1, MG571781.1
HAdV-B	NC_011203.1
HAdV-F	NC_001454.1
HBoV	AB480171.1
HSV-1	MH999839.1, NC_001806.2
EBV	NC_009334.1
CMV	NC_006273.2
HHV-7	NC_001716.2
HPV	KC470274.1, NC_001526.4, EF177191.1, NC_001593.1, MK313770.1, NC_039089.1
HBV	AY167101.1, JN406371.1, KC774376.1, LC753650.1
HIV-1	MH029899.1, MN124512.1, MT635141.1
Acinetobacter baumannii	GCF_009759685.1
Acinetobacter pittii	GCF_000369045.1

Acinetobacter seifertii	GCF_000368065.1
Chlamydia trachomatis	GCF_000012125.1
Clostridioides difficile	GCF_001077535.1
Enterococcus faecium	GCF_001544255.1
Enterococcus faecalis	GCF_000392875.1
Fusobacterium nucleatum	GCF_008633215.1
Haemophilus influenzae	GCF_001457655.1
Klebsiella aerogenes	GCF_000215745.1
Klebsiella pneumoniae	GCF_000742135.1
Metamycoplasma hominis	GCF_000085865.1
Moraxella catarrhalis	GCF_001632285.1
Morganella morganii	GCF_019243775.1
Mycobacterium tuberculosis	GCF_000195955.2
Mycoplasmoides pneumoniae	GCF_001272835.1
Pseudomonas aeruginosa	GCF_001457615.1
Staphylococcus aureus	GCF_001027105.1
Staphylococcus epidermidis	GCF_006742205.1
Stenotrophomonas maltophilia	GCF_900186865.1
Streptococcus constellatus	GCF_900459125.1
Streptococcus parasanguinis	GCF_000963275.1
Streptococcus pneumoniae	GCF_001457635.1
Ureaplasma parvum	GCF_000019345.1
Candida albicans	GCA_000182965.3
Candida glabrata	GCA_000002545.2
Candida tropicalis	GCA_000006335.3

Pneumocystis jirovecii	GCF_001477535.1

Table S6. Conserved marker genes used for taxonomic identification and phylogenetic analysis.

Taxonomy	Conserved marker gene
Acinetobacter	*gyrB*
Enterococcus	*rpoB*
Clostridioides	*rpoB*
Streptococcus	*rpoB*
Pseudomonas	*rpoB*
Candida	*TEF1*
Pneumocystis	*beta tubulin*
Metamycoplasma	*gyrB*
Mycoplasmoides	*gyrB*
Ureaplasma	*ftsH*
Mycobacterium	*rpoB*
Heamophilus	*rpoB*
Fusobacterium	*rpoB*
Moraxella	*rpoB*
Stenotrophomonas	*rpoB*
Morganella	*rpoB*
Staphylococcus	*rpoB*
Chlamydia	*rpoB*
Klebsiella	*rpoB*
SARS CoV 2	*S*
HCoV-HKU1	*S*
HCoV-229E	*S*
HCoV-OC43	*S*
SaV	*VP1*
RVA	*VP7*
HAstV	*ORF2*
HRV	*VP1*
HBV	*S*
HMPV	*F*
HPIV3	*HN-2*
H3N2	*HA*
Alphapapillomavirus	*L1*
HAdV	*Hexon*
HHV	*gB*
HCV	*NS5B*
HRSV	*G*
HBoV	*VP1*
EV	*VP1*

Table S7. Host gene sets and functional modules used for transcriptomic profiling.

Analysis Label	Geneset name	Source URL
Sample type markers (BALF)	TRAVAGLINI_LUNG_ALVEOLAR_EPITHELIAL_TYPE_1_CELL	https://www.gsea-msigdb.org/gsea/msigdb/human/geneset/TRAVAGLINI_LUNG_ALVEOLAR_EPITHELIAL_TYPE_1_CELL
Sample type markers (Throat swabs)	BUSSLINGER_ESOPHAGEAL_LATE_SUPRABASAL_CELLS	https://www.gsea-msigdb.org/gsea/msigdb/human/geneset/BUSSLINGER_ESOPHAGEAL_LATE_SUPRABASAL_CELLS
Sample type markers (Cervical swabs)	FAN_OVARY_CL0_XBP1_SELK_HIGH_STROMAL_CELL	https://www.gsea-msigdb.org/gsea/msigdb/human/geneset/FAN_OVARY_CL0_XBP1_SELK_HIGH_STROMAL_CELL
Sample type markers (Stool)	GAO_LARGE_INTESTINE_24W_C10_ENTEROCYTE	https://www.gsea-msigdb.org/gsea/msigdb/human/geneset/GAO_LARGE_INTESTINE_24W_C10_ENTEROCYTE
Sample type markers (Whole blood/serum)	HE_LIM_SUN_FETAL_LUNG_C3_DEFINITIVE_ERYTHROCYTE	https://www.gsea-msigdb.org/gsea/msigdb/human/geneset/HE_LIM_SUN_FETAL_LUNG_C3_DEFINITIVE_ERYTHROCYTE
Housekeeping geneset	HSIAO_HOUSEKEEPING_GENES	https://www.gsea-msigdb.org/gsea/msigdb/human/geneset/HSIAO_HOUSEKEEPING_GENES
Cytokine Network	BIOCARTA_CYTOKINE_PATHWAY	https://www.gsea-msigdb.org/gsea/msigdb/human/geneset/BIOCARTA_CYTOKINE_PATHWAY
Inflammatory response	HALLMARK_INFLAMMATORY_RESPONSE	https://www.gsea-msigdb.org/gsea/msigdb/human/geneset/HALLMARK_INFLAMMATORY_RESPONSE
Control marker	MANNO_MIDBRAIN_NEUROTYPES_HRGL1	https://www.gsea-msigdb.org/gsea/msigdb/human/geneset/MANNO_MIDBRAIN_NEUROTYPES_HRGL1
Antibacterial Innate Immune Response	GOBP_ANTIBACTERIAL_INNATE_IMMUNE_RESPONSE	https://www.gsea-msigdb.org/gsea/msigdb/human/geneset/GOBP_ANTIBACTERIAL_INNATE_IMMUNE_RESPONSE
Opportunistic Bacterial Infection	HP_OPPORTUNISTIC_BACTERIAL_INFECTION	https://www.gsea-msigdb.org/gsea/msigdb/human/geneset/HP_OPPORTUNISTIC_BACTERIAL_INFECTION

MHC Class II	KEGG_MEDICUS_REFERENCE_ANTIGEN_PROCESSING_AND_PRESENTATION_BY_MHC_CLASS_II_MOLECULES	https://www.gsea-msigdb.org/gsea/msigdb/human/geneset/KEGG_MEDICUS_REFERENCE_ANTIGEN_PROCESSING_AND_PRESENTATION_BY_MHC_CLASS_II_MOLECULES
MHC Class I	REACTOME_ANTIGEN_PRESENTATION_FOLDING_ASSEMBLY_AND_PEPTIDE_LOADING_OF_CLASS_I_MHC	https://www.gsea-msigdb.org/gsea/msigdb/human/geneset/REACTOME_ANTIGEN_PRESENTATION_FOLDING_ASSEMBLY_AND_PEPTIDE_LOADING_OF_CLASS_I_MHC
IFNG Response	HALLMARK_INTERFERON_GAMMA_RESPONSE	https://www.gsea-msigdb.org/gsea/msigdb/human/geneset/HALLMARK_INTERFERON_GAMMA_RESPONSE
IFNA Response	HALLMARK_INTERFERON_ALPHA_RESPONSE	https://www.gsea-msigdb.org/gsea/msigdb/human/geneset/HALLMARK_INTERFERON_ALPHA_RESPONSE
Inflammatory Response	HALLMARK_INFLAMMATORY_RESPONSE	https://www.gsea-msigdb.org/gsea/msigdb/human/geneset/HALLMARK_INFLAMMATORY_RESPONSE
IL6 Jak Stat3 Signaling	HALLMARK_IL6_JAK_STAT3_SIGNALING	https://www.gsea-msigdb.org/gsea/msigdb/human/geneset/HALLMARK_IL6_JAK_STAT3_SIGNALING
TNFa Signaling Via Nfkb	HALLMARK_TNFA_SIGNALING_VIA_NFKB	https://www.gsea-msigdb.org/gsea/msigdb/human/geneset/HALLMARK_TNFA_SIGNALING_VIA_NFKB
IL2 Stat5 Signaling	HALLMARK_IL2_STAT5_SIGNALING	https://www.gsea-msigdb.org/gsea/msigdb/human/geneset/HALLMARK_IL2_STAT5_SIGNALING
